# Genome‐wide association mapping of seedling and adult plant response to stem rust in a durum wheat panel

**DOI:** 10.1002/tpg2.20105

**Published:** 2021-06-19

**Authors:** Shitaye H. Megerssa, Mark E. Sorrells, Karim Ammar, Maricelis Acevedo, Gary C. Bergstrom, Pablo Olivera, Gina Brown‐Guedira, Brian Ward, Ashenafi G. Degete, Bekele Abeyo

**Affiliations:** ^1^ Plant Breeding and Genetics Section, School of Integrative Plant Science Cornell University Ithaca NY USA; ^2^ School of Integrative Plant Science, Plant Pathology and Plant‐Microbe Biology Section Cornell University Ithaca NY USA; ^3^ Department of Global Development Cornell University Ithaca NY USA; ^4^ International Maize and Wheat Improvement Center (CIMMYT) Mexico DF Mexico; ^5^ USDA–ARS Plant Science Raleigh NC USA; ^6^ Debre Zeit Agricultural Research Center Ethiopian Institute of Agricultural Research (EIAR) Debre Zeit Ethiopia; ^7^ International Maize and Wheat Improvement Center (CIMMYT) Addis Ababa Ethiopia; ^8^ Department of Plant Pathology University of Minnesota St. Paul MN 55108 USA

## Abstract

Many of the major stem rust resistance genes deployed in commercial wheat (*Triticum* spp.) cultivars and breeding lines become ineffective over time because of the continuous emergence of virulent races. A genome‐wide association study (GWAS) was conducted using 26,439 single nucleotide polymorphism (SNP) markers and 280 durum wheat [*Triticum turgidum* L. subsp. *Durum* (Desf.) Husnot] lines from CIMMYT to identify genomic regions associated with seedling resistance to races TTKSK, TKTTF, JRCQC, and TTRTF and field resistance to TKTTF and JRCQC. The phenotypic data analysis across environments revealed 61–91 and 59–77% of phenotypic variation was explained by the genotypic component for seedling and adult plant response of lines, respectively. For seedling resistance, mixed linear model (MLM) identified eight novel and nine previously reported quantitative trait loci (QTL) while a fixed and random model circulating probability unification (FarmCPU) detected 12 novel and eight previously reported QTL. For field resistance, MLM identified 12 novel and seven previously reported loci while FarmCPU identified seven novel and nine previously reported loci. The regions of *Sr7a*, *Sr8155B1*, *Sr11*, alleles of *Sr13*, *Sr17*, *Sr22/Sr25*, and *Sr49* were identified. Novel loci on chromosomes 3B, 4A, 6A, 6B, 7A, and 7B could be used as sources of resistance to the races virulent on durum wheat. Two large‐effect markers on chromosome 6A could potentially be used to differentiate resistant haplotypes of *Sr13* (R1 and R3). Allelism tests for *Sr13*, breaking the deleterious effect associated with *Sr22/Sr25* and retaining the resistance allele at the *Sr49* locus, are needed to protect future varieties from emerging races.

## Core Ideas

- A panel of durum wheat lines evaluated for responses to *Pgt* at the seedling and adult plant stages.
- Using 280 lines and 26,439 SNP markers GWAS models were fitted and QTL consistency was assessed.
- Genomic regions of *Sr7a*, *Sr8155B1*, *Sr11*, alleles of *Sr13*, *Sr17*, *Sr22*, *Sr25*, *Sr49* were identified.
- Novel stem rust resistance loci were identified on chromosomes 3B, 4A, 6A, 6B, 7A, and 7B.
- The identified novel loci were consistent across races, seasons, growth stages, and models.

**Abbreviations**
BLUP: best linear unbiased prediction
CI: coefficient of infection
CMLM: compressed mixed linear model
FarmCPU: fixed and random model circulating probability unification
FDR: false discovery rate
GID: genotype identification
GWAS: genome‐wide association study
IT: infection type
LD: linkage disequilibrium
LMM: linear mixed model
MLM: mixed linear model
MTA: marker–trait association
Q‐Q: quantile–quantile
QTL: quantitative trait loci
SNP: single nucleotide polymorphism

## 1 INTRODUCTION

Durum wheat [*Triticum* t*urgidum* L., ssp*. durum* (Desf.) Husnot] is a tetraploid wheat species grown in different parts of the world with the major production region being the Mediterranean Basin (Letta et al., 2013; Shewry & Hey, 2015; Kabbaj et al., 2017). Stem rust of wheat, caused by *Puccinia graminis* Pers. f. sp. *tritici* Eriks. and Henn., is among the most damaging fungal diseases of common wheat (*Triticum aestivum* L.) and durum wheat worldwide. Stem rust can occur in all wheat production areas where the environment is favorable for disease development (Singh et al., 2008). Susceptible varieties in these areas can incur a total yield loss under severe epidemics (Yu et al., 2014). The stem rust pathogen interferes with the transport of nutrients through the vascular system and results in shriveled seeds at harvest, stem breakage, and lodging (Bhavani et al., 2019). Shriveled seeds harvested from stem rust infected wheat degrade end‐use product quality (Singh et al., 2006).

Stem rust epidemics have occurred in several regions of the world at different periods and caused varying levels of yield loss (Bajgain et al., 2015a; Nirmala et al., 2017). This damage is attributed to the narrow genetic base of stem rust resistance of cultivars and breeding lines in some regions of the world (Fu & Somers, 2009; Newcomb et al., 2013). During the epidemics of stem rust in the United States, disease occurrence has been effectively controlled by the use of resistance genes in wheat cultivars (McIntosh et al., 1995) and eradication of the alternative host common barberry (*Berberis vulgaris* L.) near wheat growing areas (Kolmer et al., 1991; Jin & Singh, 2006; Singh et al., 2015; Nirmala et al., 2017). However, the emergence of new virulent races like TTKSK (Ug99) that defeated the resistance conferred by *Sr31* (Singh et al., 2011; Bajgain et al., 2015b) and other virulent races unrelated to Ug99 with broad virulence to commercially deployed resistance genes have continued to limit global production of both common and durum wheat. Race TTKSK was identified in Uganda in 1999 and spread to eastern Africa and the Middle East (Singh et al., 2006). This race with 13 variants has been recognized as a severe threat to worldwide wheat production and food security because of its broad virulence to several resistance genes mainly deployed in commercial wheat varieties and germplasm (Singh et al., 2011, 2015; Olivera et al., 2012a; Bajgain et al., 2015b; Newcomb et al., 2016; Chao et al., 2017). Race TKTTF is unrelated to the Ug99 group of races and it is predominant in Ethiopia with broad virulence to several *Sr* genes. This race caused severe yield loss during the epidemics of 2013 and 2014 and devastated the popular bread wheat ‘Digalu’ grown on over 100,000 ha (Olivera et al., 2015; Singh et al., 2015). Race TKTTF defeated the resistance conferred by *SrTmp* gene in Digalu. Pathogen races outside of the Ug99 race group and with relevant virulence on durum wheat have also been reported in the past decade. Race JRCQC is unrelated to the Ug99 lineage, and it was identified in Ethiopia in 2009. JRCQC has a combined virulence to *Sr9*e and *Sr13b*, alleles of commonly deployed resistance genes in durum wheat (Olivera et al., 2012b; Zhang et al., 2017). This race was identified upon evaluation of durum wheat germplasm from North America and CIMMYT that were mostly resistant to races in Kenya at that time but became highly susceptible when evaluated in the field nursery in Ethiopia (Olivera et al., 2012b; Singh et al., 2015). TTRTF is another virulent race on durum wheat that caused a severe epidemic on durum wheat in Sicily, Italy, after a severe epidemic in 2016 (Bhattacharya, 2017). This race was observed for the first time in Georgia in 2014 and carries broad virulence to several resistance genes in durum and common wheat including *Sr9e*, *Sr13b*, *Sr35*, *Sr36*, *Sr37*, *Sr38*, *Sr45*, and *SrTmp* (Olivera et al., 2019). A pathogen survey report from Sicily, Italy, indicated that race TTRTF is virulent on 25 durum wheat cultivars and breeding lines including major varieties grown in the region (Randazzo et al., 2016). Among the resistance genes most deployed in durum wheat in different regions of the world, *Sr13a* is still effective against the *Pgt* races virulent on durum, including TTRTF and JRCQC (Zhang et al., 2017; Olivera et al., 2019).

The stem rust pathogen evolves continuously, producing new races with virulence to resistance genes commonly deployed in commercial varieties and breeding lines. The narrow genetic base of stem rust resistance in durum wheat compared with common wheat exposes the crop to a risk of resistance being defeated by an emerging virulent race. Nevertheless, the application of genetic resistance is a preferred method to control stem rust in light of environmental safety and cost efficiency; broadening the genetic base of resistance is paramount. In an attempt to manage stem rust through the application of genetic resistance, over 60 stem rust resistant genes and alleles have been cataloged. However, most of them are major‐effect gene resistances (*R* genes) which are most often effective against specific races (McIntosh et al., 1995, 2017). Therefore, the continuous evaluation and identification of new sources of resistances to stem rust, characterization of the available sources of resistance in the germplasm pool and their proper use is crucial to mitigate the risk posed by stem rust on global wheat production. Although there is a possibility of incorporating novel sources of resistances in breeding materials from wild relatives or landraces, breaking the linkage drag is often challenging. The current study uses a panel of breeding lines from CIMMYT to evaluate and characterize sources of resistance to virulent races of the stem rust pathogen through association mapping.

Association mapping (linkage disequilibrium [LD] mapping) is an efficient approach to identify marker–trait associations (MTAs) (Zhu et al., 2008). This technique exploits genetic recombination that occurred over generations in the population used for study (Zhu et al., 2008; Chao et al., 2017) and is a powerful method for studying simple and complex traits in many crop species (Kushwaha et al., 2017). Mapping resolution is higher in association mapping than linkage mapping because of a higher level of polymorphism on using a population comprised of diverse lines. However, population structure must be considered in genome‐wide association study (GWAS) analysis models if the population under study has a stratification which otherwise can result in false positive associations (Yu & Buckler, 2006).

Genetic studies to identify and map sources of stem rust resistance in durum wheat using dense marker coverage is limited compared with that of common wheat. Moreover, the panel of CIMMYT durum wheat lines used in the current study have not previously been evaluated for seedling response to TTKSK, TKTTF, the durum virulent races (JRCQC and TTRTF), or field response against single races. Therefore, the objectives of the current study were to (a) evaluate seedlings of a panel of durum wheat lines for resistance to four virulent *Pgt* races (TTKSK, JRCQC, TKTTF, and TTRTF) and field resistance to races JRCQC and TKTTF and (b) conduct GWAS analysis using single nucleotide polymorphism (SNP) markers to identify genomic regions associated with seedling and field resistances against these races.

**Core Ideas**

- A panel of durum wheat lines evaluated for responses to *Pgt* at the seedling and adult plant stages.
- Using 280 lines and 26,439 SNP markers GWAS models were fitted and QTL consistency was assessed.
- Genomic regions of *Sr7a*, *Sr8155B1*, *Sr11*, alleles of *Sr13*, *Sr17*, *Sr22*, *Sr25*, *Sr49* were identified.
- Novel stem rust resistance loci were identified on chromosomes 3B, 4A, 6A, 6B, 7A, and 7B.
- The identified novel loci were consistent across races, seasons, growth stages, and models.

## 2 MATERIALS AND METHODS

### 2.1 Plant materials and phenotyping

#### 2.1.1 Seedling evaluation

A panel of 283 spring durum wheat lines representing the germplasm pool of the CIMMYT durum wheat breeding program was evaluated against four *Pgt* races in a biosafety level‐3 greenhouse facility at the University of Minnesota in January 2019. The four races were TTKSK (isolate 04KEN156/04), JRCQC (isolate 09ETH08‐1), TKTTF (isolate 13ETH18‐1), and TTRTF (isolate 14GEO189‐1). These races were selected based on their broad virulence on commercially deployed resistance genes and their damage on global wheat production. Six seeds of each line were planted in trays filled with vermiculite and replicated twice for each race. Seven‐day‐old seedlings were inoculated with urediniospores of each race following the procedure by Rouse et al. (2011). Seedlings were scored 14 d postinoculation using the 0‐to‐4 scale described by Stakman et al. (1962). Accordingly, infection types (ITs) “;”, “0”, “1^–^”, “1”, “1^+^”, “2^–^”, “2”, and “2^+^” were considered resistant whereas “3^–^”, “ 3”, “ 3^+^”, and “4” were considered susceptible. This scale was linearized to 0‐to‐9 scale according to Zhang et al. (2011) as ‘;’ and ‘0’ = 0, ‘1^–^’ = 1, ‘1’ = 2, ‘1^+^’ = 3, ‘2^–^’ = 4, ‘2’ = 5, ‘2^+^’ = 6, ‘3^–^’ = 7, ‘3’ = 8, ‘3^+^’ = 9, ‘4’ = 9 for statistical analysis. Lines with linearized scale ≤6 (IT ≤ 2^+^) and >6 (IT > 2^+^) were considered seedling resistant and susceptible, respectively.

#### 2.1.2 Adult plant evaluation

The same panel used for seedling evaluation was tested for responses to races TKTTF and JRCQC at the adult plant stage in single‐race nurseries at the Debre Zeit Agricultural Research Center, Ethiopia, from 2018 to 2020. The response to race JRCQC was evaluated during main‐season 2019 (JRCQC_MS19) and off‐season 2020 (JRCQC_OS20), while that of race TKTTF was evaluated during the main‐season 2018 (TKTTF_MS18) and main‐season 2019 (TKTTF_MS19). The TKTTF_MS18 nursery was inoculated with bulk of isolates ETH‐9TZaTX25, SR‐BA‐14, SR‐BA‐28, AM‐S, AM‐14, AM#‐a1, Am‐03, while TKTTF_MS19 was inoculated with bulk of isolates AM‐A4, Am‐A17, AM‐B28, DZ‐A‐8, DZ‐A25, and Gonder‐A‐2. The JRCQC_MS19 and JRCQC_MS20 trials were inoculated with bulk of isolates Ku#3, Ku#22, Ku#30, Am#6, and BD#30 identified in 2015 and 2016. The main‐ and off‐seasons in Ethiopia are from June to November and from January to May, respectively. The nurseries were established in isolation from the international screening nursery, where germplasm screening is done against a bulk of multiple races, to avoid potential contamination. Moreover, the two single‐race nurseries were also isolated by distance (∼1 km apart) to control contamination. The lines were planted in double rows (1 by 0.2 m) using a randomized incomplete‐block design and two replications. One moderately resistant (‘Mangudo’) and two susceptible (‘Local Red’ and ‘Arendato’) checks were planted after every 50 lines. The 20 stem rust differential lines were planted at the start and end of each nursery. The cultivar Leeds, carrying *Sr13/Sr13b* and cultivar Digalu carrying *SrTmp* were planted perpendicular to the plots and surrounding the nursery as spreader rows to initiate infections on the trials of JRCQC and TKTTF, respectively. Moreover, the nurseries were surrounded by oat (*Avena sativa* L.) (a nonhost for *Puccinia graminis* f.sp. *tritici*) to act as a physical barrier to potential spore contaminations. Spores of the bulk isolates of each race were mixed with distilled water and a drop of Tween 20 was added to reduce surface tension of water (one drop per 0.5 L). Each nursery was inoculated twice with this mixture at stem elongation (Zadok's growth stage = 31) (Zadoks et al., 1974).

Disease severity was scored according to the modified Cobb's scale by estimating the proportion of the stem area (0–100%) covered by rust pustules (Peterson et al., 1948). Infection response was scored according to Roelfs et al.(1992) based on the size of pustules and amount of chlorosis and necrosis on the stem. The responses classes are ‘0’ for no visible infection, ‘R’ for resistant, ‘MR’ for moderately resistant, ‘MS’ for moderately susceptible, and ‘S’ for susceptible. The nursery was scored three times for JRCQC_MS19 and TKTTF_MS19 and four times for TKTTF_MS18 and JRCQC_OS20. The severity and response were combined to a coefficient of infection (CI) value by multiplying the severity with a 0‐to‐1 scale assigned for each response class. The scale was assigned as follows: immune = 0.0, R = 0.2, MR = 0.4, MS = 0.8, and S = 1.0. The mean of the scale of responses was used to calculate CI in the cases where combinations of infection responses were scored for a given genotype (Stubbs et al., 1986). Then, the CI was used for further statistical analysis and the last scoring was considered to calculate the CI in all except TKTTF_MS18, where the third scoring was used.

### 2.2 Statistical analysis of phenotype data

#### 2.2.1 Seedling response

The linearized scale of the seedling response against the four races was used to apply statistical analysis. R statistical software Version 3.6.1 (R Core Team, 2020) was used to plot the distributions of the responses and analyze the correlation between responses against the four races. A linear mixed model (LMM) described in Equation 1 was fitted using the lmer() function of the R package *lme4* (Bates et al., 2015) considering the genotype and replication as random:

(1) $$y_{ij}=\mu +g_{i}+r_{j}+\varepsilon_{ij}$$

where *y_ij_* is the response of the *i*th line at the *j*th replication, μ is the overall mean response, *g_i_* is the random effect of the *i*th genotype (line), *r_j_* is the random effect of the *j*th replication, and ε*_ij_* is the residual associated with the model. Variance components estimated from Equation 1 above were used to calculate broad‐sense heritability (*H* ^2^) Holland et al. (2003):

(2) $$H^{2}=\frac{V_{g}}{V_{p}}$$

where *H* ^2^ is the broad‐sense heritability, *V*_g_ is the variance resulting from the genotype (line), V_p_ is the variance resulting from the phenotype, *Vp* = *Vg + Ve*, and *V*_e_ is the residual variance. The race × genotype (line) effect was estimated from LMM described in Equation 3 using the lmer() function of R considering genotype or line, race, and line × race interaction as random effects:

(3) $$y_{ijk}=\mu +g_{i}+r_{j}+{\left(gr\right)}_{ij}+R_{k}+\varepsilon_{ijk}$$

where *y_ijk_* is the response of the *i*th line in the *j*th race and *k*th replication, μ is the overall mean response, *g_i_* is the random effect of the *i*th genotype (line), *r_j_* is the random effect of the *j*th race, gr*_ij_* is the interaction effect of the *i*th line and the *j*th race as random, *R_k_* is the random effect of the *k*th replication, and ε*_ijk_* is the residual associated with the model. The variance components estimated from Equation 3 was used to calculate broad‐sense heritability (*H* ^2^) (Tsilo et al., 2014):

(4) $$H^{2}=\frac{V_{g}}{V_{g}+\frac{V_{gr}}{n(r)}+\frac{V_{e}}{n(r)\times n(\mathrm{rep})}}$$

where *H* ^2^ is broad‐sense heritability, *V*_g_ is the variance as a result of the genotype (line), *V*_gr_ is the variance as a of the genotype × race interaction, *V*_e_ is the variance as a result of the error (residual), *n*(*r*) is number of races, and *n*(rep) is number of replications.

#### 2.2.2 Adult plant response

The LMM was fitted on the CI as a response variable for the JRCQC_MS19, TKTTF_MS19 and JRCQC_OS20, while the square root transformed CI was used for TKTTF_MS18. For JRCQC_MS19 and TKTTF_MS19, the following model (Equation 5) was fit using the lmer() function of the R package *lme4* to estimate the variance components:

(5) $$y_{ijk}=\mu +g_{i}+C_{j}+r_{k}+\varepsilon_{ijk}$$

where *y_ijk_* is the response of the *i*th line in the *j*th column and the *k*th replication, *g_i_* is the random effect of the *i*th line, *C_j_* is the fixed effect of the *j*th column, *r_k_* is the random effect of *k*th replication, and ε*_ijk_* is the residual associated with the model.

For TKTTF_MS18 and JRCQC_OS20, the models described in Equation 6 and Equation 1, respectively, were fit using ASReml‐R (Gilmour et al., 2009) to estimate the variance components. Best linear unbiased predictions (BLUPs) were calculated from the respective models and the broad‐sense heritability was calculated using Equation 2 for each race across seasons. The variable *R_j_* in Equation 6 is the fixed effect of the *j*th row, and the remaining descriptions were same as Equation 5:

(6) $$y_{ijk}=\mu +g_{i}+R_{j}+r_{k}+\varepsilon_{ijk}$$

#### 2.2.3 Genotyping, population structure, and LD analyses

The same panel of 283 lines from the CIMMYT durum wheat breeding germplasm pool used for adult plant evaluation against multiple races in eastern Africa (Ethiopia and Kenya) was genotyped using genotyping‐by‐sequencing following the protocol described by Poland et al. (2012). Single nucleotide polymorphism genotype calling, data filtering, and data imputation were performed as described in Megerssa et al. (2020) on a GWAS study of the same panel for response to bulk of multiple *Pgt* races prevalent in eastern Africa. A total of 26,439 SNP markers for 280 lines were retained for GWAS analysis. The LD between pairs of SNPs was calculated as the squared allele frequency correlation (*r* ^2^) using TASSEL software version 5 (Bradbury et al., 2007) as described in Megerssa et al. (2020). The presence of population structure was assessed using principal component analysis. The extent of LD and population structure was previously reported for this panel (Megerssa et al., 2020).

#### 2.2.4 Genome‐wide association study analysis

Genome‐wide association study analysis was conducted using GAPIT (Lipka et al., 2012) by fitting three models: mixed linear model (MLM) (Yu et al., 2006), compressed MLM (CMLM) (Zhang et al., 2010), and fixed and random model circulating probability unification (FarmCPU) (Liu et al., 2016). The mean linearized scale of the two reps for the seedling response to the four races and the BLUPs calculated from the respective models for the adult plant response against the two single races (JRCQC and TKTTF) were used as a response in the fitted GWAS models. The first two principal component analysis scores and the kinship matrix were fitted as fixed and random effects, respectively.

The results of GWAS were visualized using Manhattan and quantile–quantile (Q‐Q) plots produced using the R package *qqman* (Turner, 2017) applied on the −log10 *P* value. The three models were compared based on the deviation of the distribution of the observed −log10 *P* value from the expected value in the Q‐Q plots and results were interpreted from MLM and FarmCPU. Significant markers on the same chromosome were grouped into QTL based on their LD. A false discovery rate (FDR) of 5% was used for multiple comparison adjustment and as a threshold to declare significant MTAs (Benjamini & Hochberg, 1995). GAPIT calculates the FDR adjusted *P* values and markers with *P* values < .05 were taken as significant MTAs. The FDR threshold value was calculated using a vector of the *P* values from the GWAS output sorted from the most significant to the least. Then using a function formed in R, a cutoff was calculated for each test using the following formula: cutoff = (1:*N*)/*N*)×FDR, where *N* was the total number of tests (No. of markers). Then the numbers of significant markers (*n*) (*P* values < .05) with the numbers of tests (*N*) and FDR threshold (0.05) were used to calculate the threshold value using the following formula: FDR threshold value = (0:*N*/*N*)×FDR(*n*+1). The −log10(threshold value) was used to mark the threshold line on the Manhattan plot. Consistent MTAs between races and race or seasons in the field were visualized using the R package *Venndiagram* (Chen & Boutros, 2011). Markers reported in previous QTL mapping studies on durum and common wheat were gathered, and their sequences were searched from the GrainGenes database. The fasta file of the sequences was searched using the BLASTn program of the IWGSC database. Then the alignment of physical positions of the significant markers identified in the current study with the chromosomal positions of the ‘Svevo’ reference assembly were compared and resistance genes and alleles were proposed based on the similarity of positions and race specificity of known stem rust resistance genes and alleles.

## 3 RESULTS

### 3.1 Phenotypic data analysis

#### 3.1.1 Seedling response to the four races

We evaluated a panel of lines representing the durum wheat breeding germplasm pool of CIMMYT for seedling responses to four *Pgt* races virulent to durum wheat. The distributions of the seedling response of the lines against the four *Pgt* races was skewed toward the resistant scores (linearized response ≤ 6 or IT ≤ 2^+^) (Supplemental Figure S1). The percentage of resistant lines varied from 56.4% against race TTRTF to 73% against race TKTTF (Table 1). Moreover, the lines exhibited resistance to combinations of races that ranged from 50.9 to 58.3% for combinations of three races and from 52.3 to 67.1% for combinations of two races (Table 2).

**TABLE 1:** Summary of the percentage resistant and susceptible lines against the four *Pgt* races and broad‐sense heritability of seedling response. Values are percentages and counts in parenthesis

Race	Resistant	Susceptible	Heritability (*H* ^2^)
	% (count)		
TTKSK	70.6 (197)	29.4 (82)	0.86
TKTTF	73.1 (204)	26.9 (75)	0.91
JRCQC	67.1 (188)	32.8 (92)	0.90
TTRTF	56.4 (159)	43.6 (123)	0.61

**TABLE 2:** Number and percentage of lines resistant at the seedling stage against different combinations of the four races

Race combination	Total no. lines	No. of resistant lines	Percentage of resistant lines
			%
TTKSK+TKTTF+JRCQC+TTRTF	283	142	50.2
TTKSK+TKTTF+JRCQC	283	165	58.3
TTKSK+JRCQC+TTRTF	283	144	50.9
TTKSK+TKTTF+TTRTF	283	145	51.1
JRCQC+TKTTF+TTRTF	283	148	52.3
TTKSK+TKTTF	283	190	67.1
TTKSK+JRCQC	283	168	59.4
TTKSK+TTRTF	283	148	52.3
JRCQC+TKTTF	283	176	62.2
TKTTF+TTRTF	283	151	53.4
JRCQC+TTRTF	283	151	53.4

**FIGURE 1:**
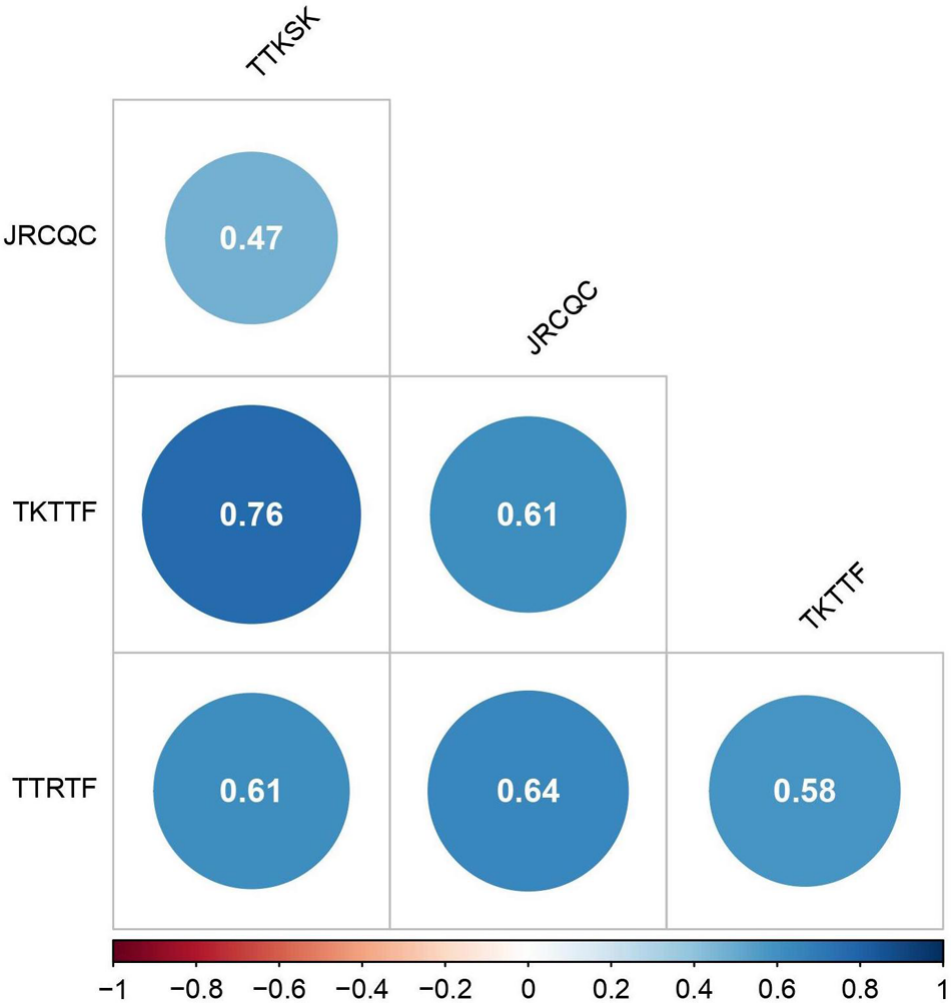
Correlation between seedling responses of durum wheat lines against four races. Large circle indicates the magnitude of the correlation while dark blue color indicates the strength (intensity) of the correlation

Of the lines evaluated, 50.2% (142 lines) were resistant to all four races, while 19.4% (55 lines) were susceptible to all the four races. Based on the infection type and race specificity, 8.6% of the lines (24 lines) were postulated to carry *Sr13b*. These lines showed low infection types for response to TTKSK (2^–^) and TKTTF (2^–,^ to 2^+^), while high infection type was scored for response to JRCQC and TTRTF (3 to 4) (Supplemental Table S1). One line (genotype identification [GID] 7147182) and two lines (GID 7147179 and 7147180) showed an immune seedling response against all four races and three races (TTKSK, TKTTF, JRCQC), respectively. The broad‐sense heritability for seedling responses to the four races varied from 0.61 for race TTRTF to 0.91 for race TKTTF (Table 1). The phenotypic correlation coefficients between the responses to the four races ranged from moderate (*r* = 0.47) between JRCQC and TTKSK to high (*r* = 0.76) between TKTTF and TTKSK (Figure 1).

#### 3.1.2 Adult plant response to the two races

The panel of lines were evaluated for field responses against two races (JRCQC and TKTTF) for two seasons from main‐season 2018 to off‐season 2020. The frequency distribution of the CI of lines was normal for JRCQC_MS19, JRCQC_OS20, and TKTTF_MS19 but skewed toward resistance for TKTTF_MS18 (Supplemental Figure S2). The normality of the CI for TKTTF_MS18 was improved after square root transformation and the transformed CI was used for further analysis. The broad‐sense heritability for the adult plant responses ranged from 0.59 for JRCQC_OS20 to 0.77 for TKTTF_MS19 (Table 3). Moderate correlations were observed between seedling and field responses to the two races (0.37–0.53 for JRCQC and 0.55–0.61 for TKTTF) (Data not shown).

**TABLE 3:** Summary of descriptive statistics, genetic variance (*V*_g_), and broad‐sense heritability (*H* ^2^) of coefficient of infection for field responses to races JRCQC and TKTTF across seasons

Statistic	JRCQC_MS19	JRCQC_OS20	TKTTF_MS18	TKTTF _MS19
Mean	36.3	39.0	23.5	38.3
Range	0–70	0–80	0–80	0–70
*V*_g_	154.8	207.9	3.4	227.1
*H* ^2^	0.67	0.59	0.74	0.77

### 3.2 Genome‐wide association study analysis

Marker–trait association analysis for seedling responses to the four *Pgt* races (TTKSK, TKTTF, JRCQC, and TTRTF), and field responses to the two single races (JRCQC and TKTTF) were conducted using GAPIT by fitting three different models (MLM, CMLM, and FarmCPU). The Q‐Q plots of MLM and FarmCPU fitted the data well for all race–season combinations and results were interpreted from these two models.

#### 3.2.1 GWAS for seedling response to the four *Pgt* races

**FIGURE 2:**
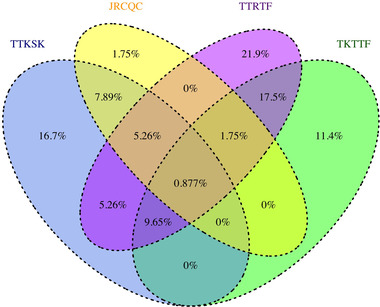
Percentage of common significant markers among seedling responses of lines against four *Pgt* races identified using mixed linear model (MLM)

The mean linearized scale of the two replications for the seedling responses of lines against the four races was used as a response variable for GWAS analysis. A total of 114 significant markers distributed along the 14 chromosomes and unaligned contigs were identified for seedling resistance against the four *Pgt* races using MLM (Supplemental Table S2). Among those, 1, 16.6, 30.7, and 51.7% were associated with seedling resistance against the four races, three of the four races, two of the four races, and a single race, respectively (Figure 2).

**FIGURE 3:**
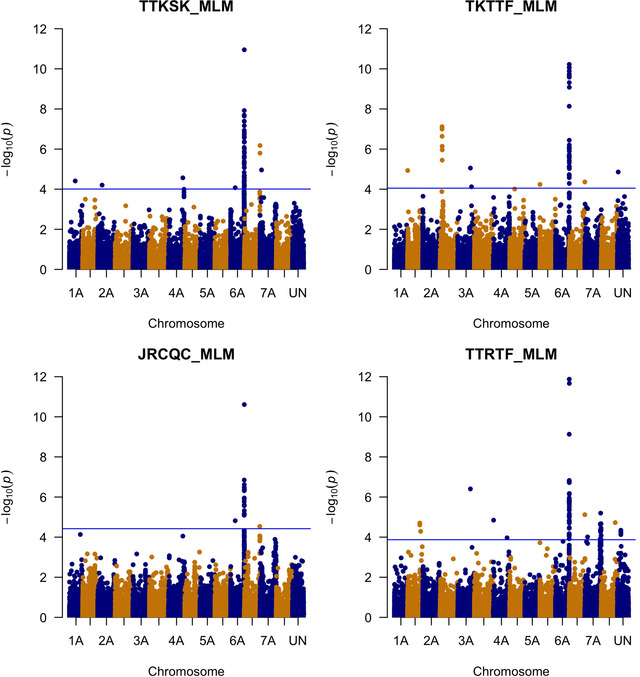
Manhattan plots of genome‐wide association study for seedling response of durum wheat lines against four *Pgt* races identified using mixed linear model (MLM)

**FIGURE 4:**
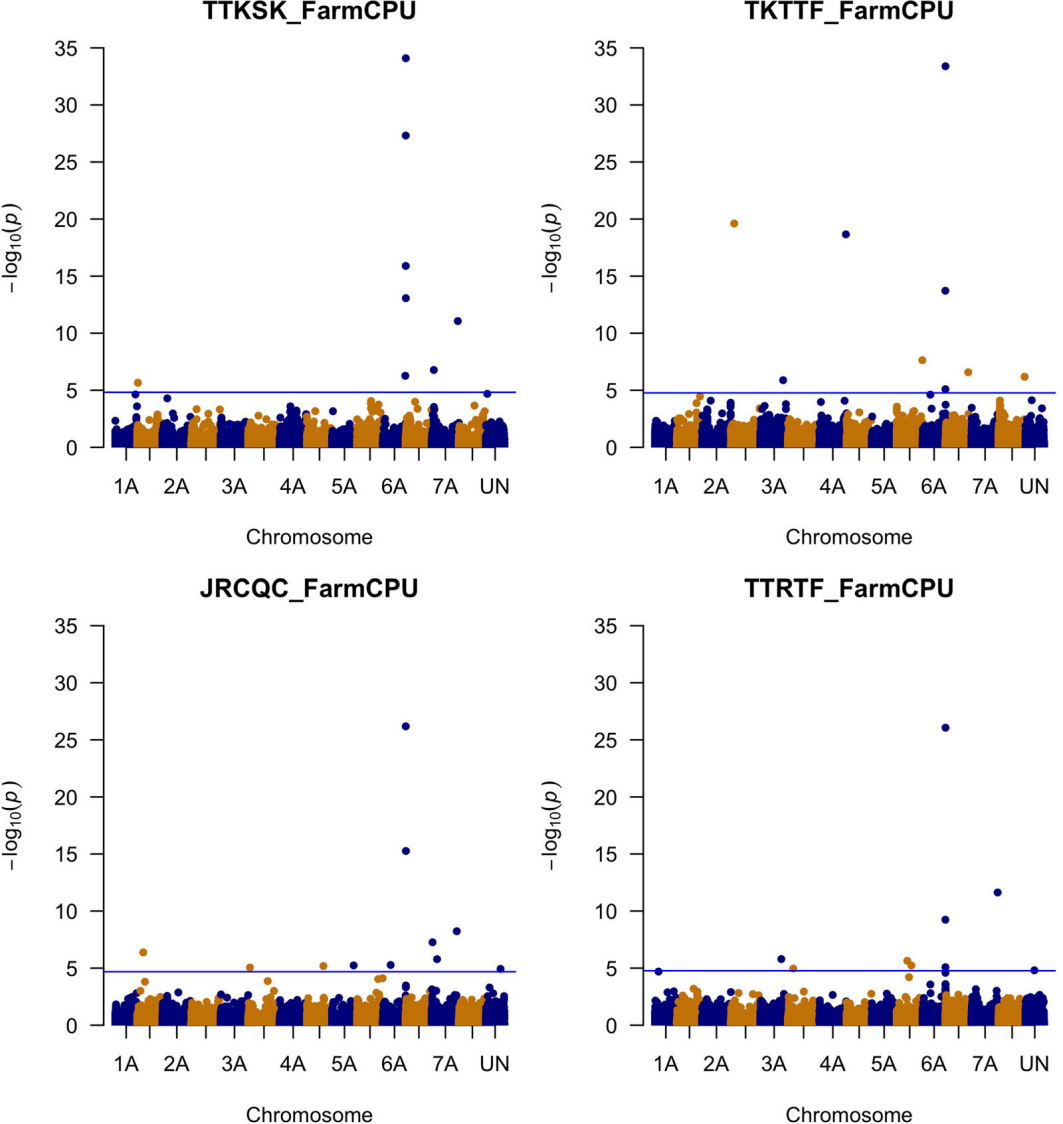
Manhattan plots of genome‐wide association study for seedling response of durum wheat lines against four *Pgt* races identified using fixed and random model circulating probability unification (FarmCPU)

Five of the MTAs were on unaligned contigs and the remaining 109 were grouped into 17 QTL represented by single and multiple adjacent markers with known chromosomal locations (Supplemental Table S2; Figure 3). The numbers of QTL identified using MLM were six, seven, two, and eight for seedling resistance against races TTKSK, TKTTF, JRCQC, and TTRTF, respectively. This study is the first to report GWAS analysis of durum wheat for response to race TTRTF. FarmCPU identified 34 significant MTAs that were grouped into 20 QTL with known chromosomal locations (Figure 4; Supplemental Table S3). Among the 34 MTAs, a single marker for each was associated with seedling resistance against combinations of two and three races, while 32 markers were associated with seedling resistance to single races. Six QTL located on chromosomes 2B (89–97 Mb), 3A (565 and 614 Mb), 6A (205 Mb and 602–615 Mb), and 7A (686–721 Mb) were consistent between the two models (Table 4).

**TABLE 4:** Lists of consistent significant markers between mixed linear model (MLM) and fixed and random model circulating probability unification (FarmCPU) for seedling resistance against four races and field resistance against the two races across seasons

Type of resistance	Position	Chromosome	Trial
	bp		
Seedling resistance	89,523,302	2B	TKTTF
	565,464,709	3A	TKTTF, TTRTF
	614,332,431	3A	TKTTF
	205,649,407	6A	TTKSK, JRCQC
	609,635,640	6A	TTKSK, TKTTF, JRCQC, TTRTF
	611,495,915	6A	TTKSK, TKTTF, JRCQC, TTRTF
	612,003,938	6A	TTKSK, TKTTF, TTRTF
	612,043,936	6A	TTKSK, TKTTF, TTRTF
	612,802,438	6A	TTKSK, TKTTF, TTRTF
	613,131,839	6A	TTKSK, TKTTF, TTRTF
	613,294,106	6A	TTKSK, TTRTF
	613,748,730	6A	TTKSK, TKTTF, TTRTF
	615,619,215	6A	TTKSK, TTRTF, JRCQC
	697,030,516	7A	TTRTF, TTKSK
	700,805,183	7A	TTRTF, TTRTF
Field resistance	689,821,784	5B	TKTTF_MS19, JRCQC_MS19, JRCQC_OS20
	615,604,035	6A	TKTTF_MS18, TKTTF_MS19, JRCQC_MS19, JRCQC_OS20
	700,805,183	7A	TKTTF_MS18, TKTTF_MS19, JRCQC_MS19, JRCQC_OS20
	717,518,884	7A	TKTTF_MS18, TKTTF_MS19, JRCQC_MS19 JRCQC_OS20

On chromosome 1A, an MTA was identified at 258 Mb for seedling response to race TTKSK (Supplemental Table S1). On chromosome 1B, six significant markers representing five putative QTL were identified (Figures 3 and 4). The 11 Mb locus was associated with seedling resistance to race TKTTF, while the regions at 550, 551, and 587 Mb were associated with seedling resistance to race TTRTF (Figure 3; Supplemental Table S2). The markers at 550 and 551 Mb were in strong LD (*r* ^2^ = 0.95) and represent the same QTL that explained 5.1% of the phenotypic variation on average (Supplemental Table S2). The remaining two MTAs, at 22 and 166 Mb identified by FarmCPU, were associated with seedling resistance to races TTKSK and JRCQC, respectively (Supplemental Table S3).

On chromosome 2B, a QTL represented by eight significant markers spanning from 89 to 97 Mb (LD, *r* ^2^ = 0.81–0.98) was identified for seedling resistance against race TKTTF (Figure 3; Supplemental Table S2). This QTL was consistent between MLM and FarmCPU, and it explained 4.2–5.8% of the phenotypic variation (Table 4).

On chromosome 3A, two MTAs consistent between the MLM and FarmCPU models were identified at 565 and 614 Mb regions. The 565 Mb locus was associated with seedling resistance to races TKTTF and TTRTF and explained 3.9 and 7.4% of the phenotypic variation, respectively, while the 614 Mb region was identified for seedling resistance to race TKTTF and explained 3.1% of the phenotypic variation (Supplemental Tables S2 and S3; Table 4). On chromosome 3B, significant associations were identified using FarmCPU at 40 and 139 Mb (FDR adjusted *p* value = .04) regions for resistance against races JRCQC and TTRTF, respectively (Supplemental Table S3).

Four significant markers (17, 619, 651, and 718 Mb) were identified on chromosome 4A (Supplemental Tables S2 and S3; Figures 3 and 4). The MTAs at 17 and 619 Mb were identified using MLM for seedling resistance against race TTRTF and explained 5.3 and 4.2% of the phenotypic variation, respectively. The 651 Mb region was associated with seedling resistance to race TTKSK and explained 5.2% of the phenotypic variation. The 718 Mb locus was detected by FarmCPU for seedling resistance against race TKTTF. On chromosome 4B, one MTA (444 Mb) was identified using FarmCPU for seedling resistance to race JRCQC (Supplemental Table S3).

On chromosome 5A, a significant marker (581 Mb) was identified for seedling resistance to race JRCQC using FarmCPU (Supplemental Table S3; Figure 4). On chromosome 5B, MTAs were detected at 287 and 396 Mb using FarmCPU for seedling resistance against race TTRTF (Supplemental Table S3; Figure 4), while two MTAs, at 61 and 691 Mb were identified for seedling resistance against race TKTTF using MLM and FarmCPU, respectively (Supplemental Tables S2 and S3).

**FIGURE 5:**
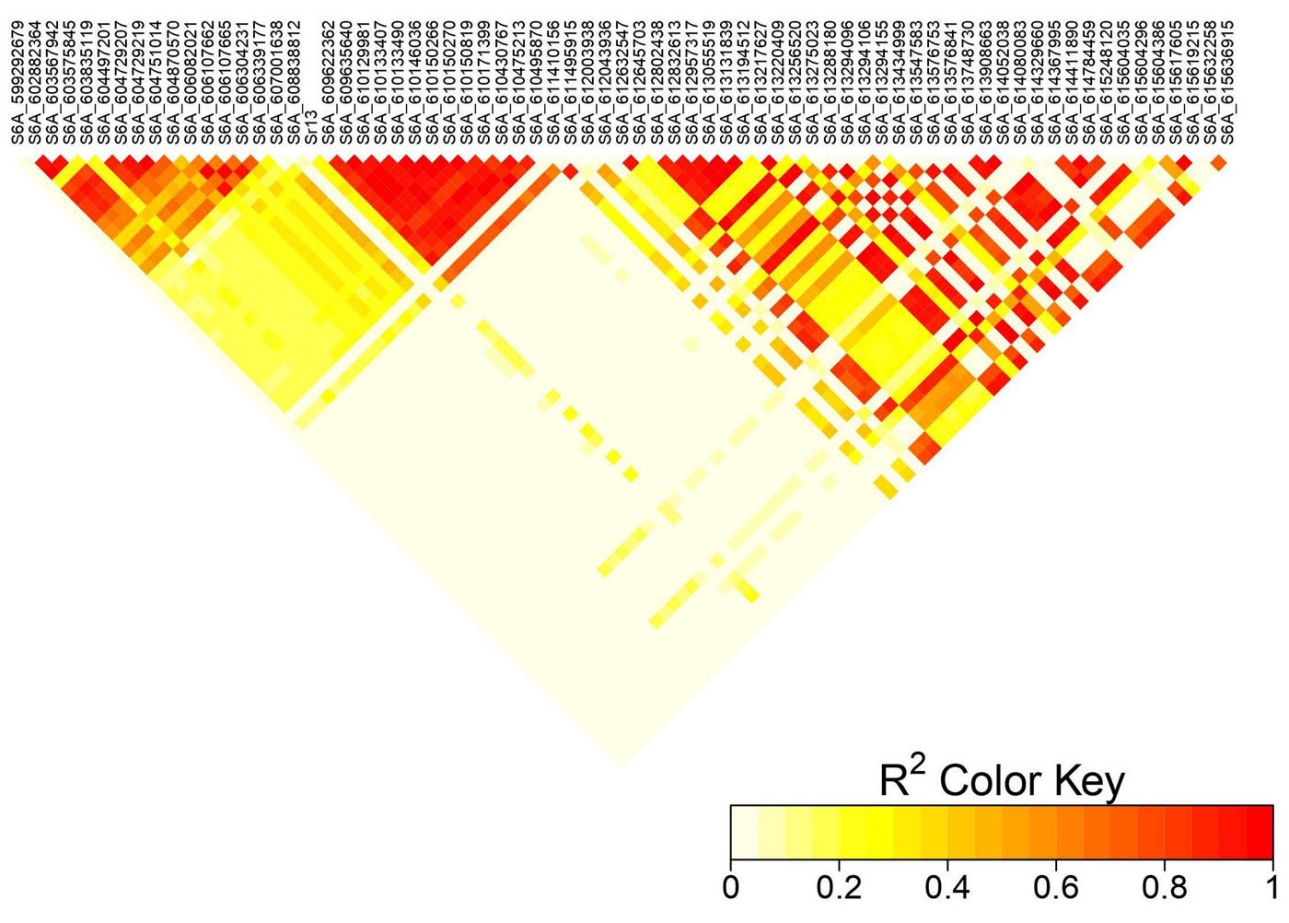
Linkage disequilibrium heatmap of significant markers on chromosome 6A identified using mixed linear model (MLM) and fixed and random model circulating probability unification (FarmCPU) for seedling resistance against four *Pgt* races and field resistance against two races

Chromosome 6A had the highest number of significant markers (70 markers) with the largest contribution to phenotypic variation (Supplemental Tables S2 and S3; Figures 3 and 4). These MTAs were identified using MLM and FarmCPU and grouped into two QTL based on their position and LD. A QTL at 205 Mb identified by both models explained 4.6% of the phenotypic variation for seedling responses to races TTKSK and JRCQC (Supplemental Tables S2 and S3). The significant markers that extended from 602 to 615 Mb may represent a single QTL. The phenotypic variation explained by these markers ranged from 4.5 to 14.5% for race TTKSK, 3.2 to 8.8% for race TKTTF, 4.9 to 11.5% for race JRCQC, and 4.2 to 17.1% for race TTRTF. A marker at 611 Mb (611,495,915 bp) was associated with seedling resistances to all four races and was detected by both MLM and FarmCPU (Table 4; Supplemental Tables S2 and S3). This marker (611 Mb) contributed the most to the phenotypic variation for the seedling response of lines to races TTKSK (*R* ^2^ = 14.5%) and JRCQC (*R* ^2^ = 11.5%) (Supplemental Table S2). Moreover, the 611 Mb marker was in weak to strong LD (*r* ^2^ = 0.13–0.75) with the significant markers extending from 602 to 610 Mb except one at 608 Mb (Figure 5). Markers at 612 Mb (612,832,613 bp) and 613 Mb (613,131,839 bp) contributed the most to the phenotypic variation for the seedling response to races TKTTF (*R* ^2^ = 8.8%) and TTRTF (*R* ^2^ = 17.1%), respectively (Supplemental Table S1). These two markers were consistent between these two races and were in strong LD (*r* ^2^ = 0.94). They were in weak to strong LD (*r* ^2^ = 0.12–0.98) with 36 significant markers extending from 612 to 615 Mb (Figures 2 and 5). All the significant markers on chromosome 6A extending from 602 to 615 Mb, except 21 markers, were in weak to moderate LD with the *Sr13* marker (*r* ^2^ = 0.10–0.40) (Figure 5). On chromosome 6B, five significant MTAs representing three putative QTL were identified (Supplemental Tables S2 and S3). A QTL tagged by two markers at 698 Mb (LD, *r* ^2^ = 0.93), identified using MLM, was associated with seedling resistance to race TTKSK and explained 7.2% of the phenotypic variation on average. A region at 693 Mb, identified using MLM for seedling resistance against races TKTTF and TTRTF, explained 3.3 and 5.7% of the phenotypic variation, respectively (Supplemental Table S2). An MTA at 609 Mb was detected using FarmCPU for seedling resistance to TKTTF (Supplemental Table S3).

**FIGURE 6:**
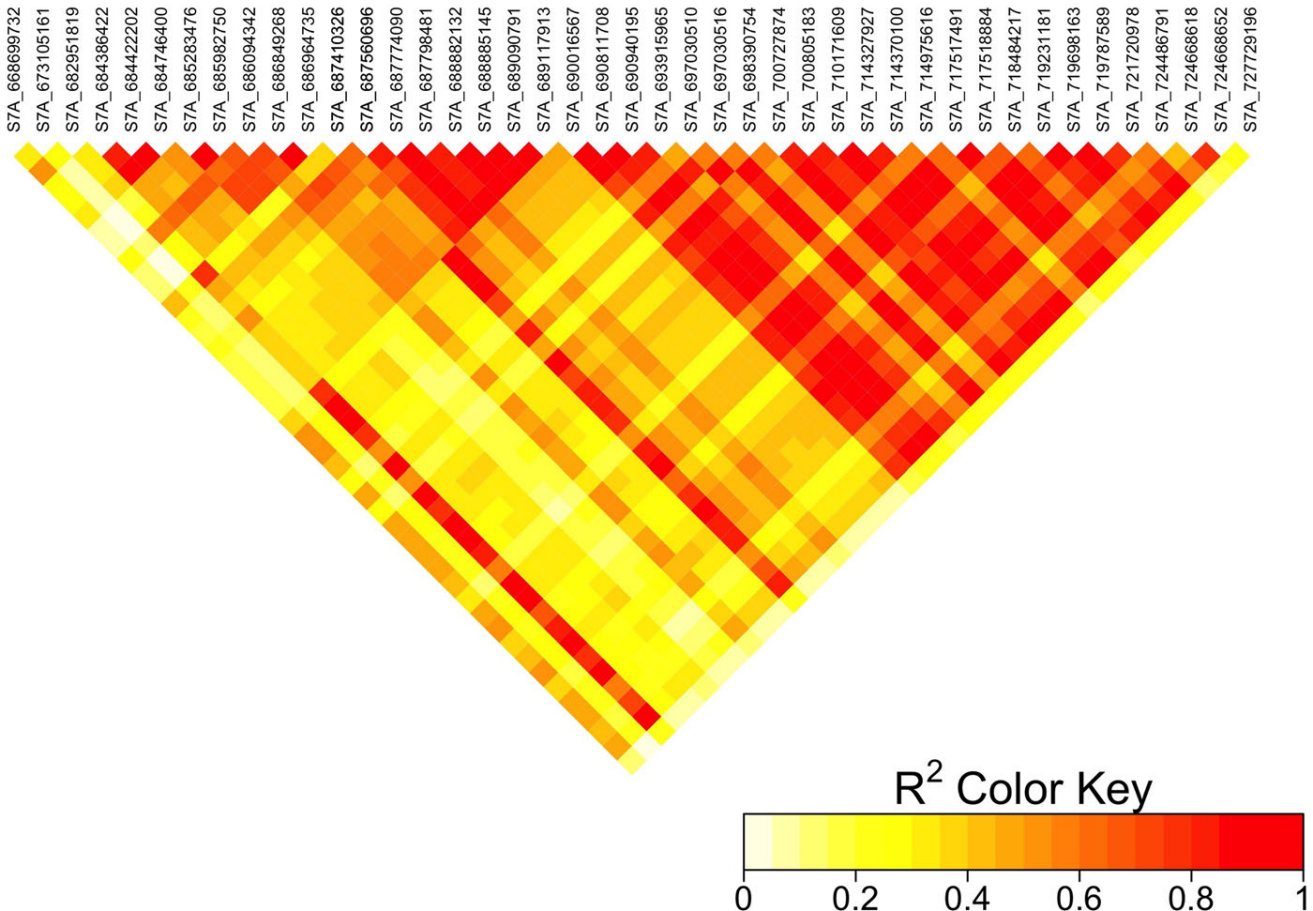
Linkage disequilibrium heatmap of significant markers on chromosome 7A identified using mixed linear model (MLM) and fixed and random model circulating probability unification (FarmCPU) for seedling resistance against four *Pgt* races and field resistance against two races

On chromosome 7A, 19 significant markers representing five putative QTL were identified using MLM and FarmCPU (Supplemental Tables S2 and S3; Figures 3 and 4). Four of the QTL represented by single markers were associated with seedling resistance to races TTKSK (51 and 67 Mb) and JRCQC (17 and 139 Mb). The fifth QTL represented by 14 significant markers extending from 668 to 721 Mb was associated with seedling resistance to races TTKSK, JRCQC, and TTRTF. These 14 markers were in moderate to strong LD (*r* ^2^ = 0.29–0.98) and explained 3.3–5.8% of the phenotypic variation (Figure 6). On chromosome 7B, significant MTAs were identified for seedling resistance against races TTRTF at 622 Mb using MLM and TKTTF at 698 Mb using FarmCPU (Supplemental Tables S2 and S3). For race JRCQC, MLM identified the QTL on chromosomes 6A only (Supplemental Table S2; Figure 3), while FarmCPU identified additional QTL on chromosomes 1B, 3B, 4B, 5A ,and 7A, albeit represented by single markers (Figure 4; Supplemental Table S3).

#### 3.2.2 GWAS for field response to JRCQC and TKTTF

**FIGURE 7:**
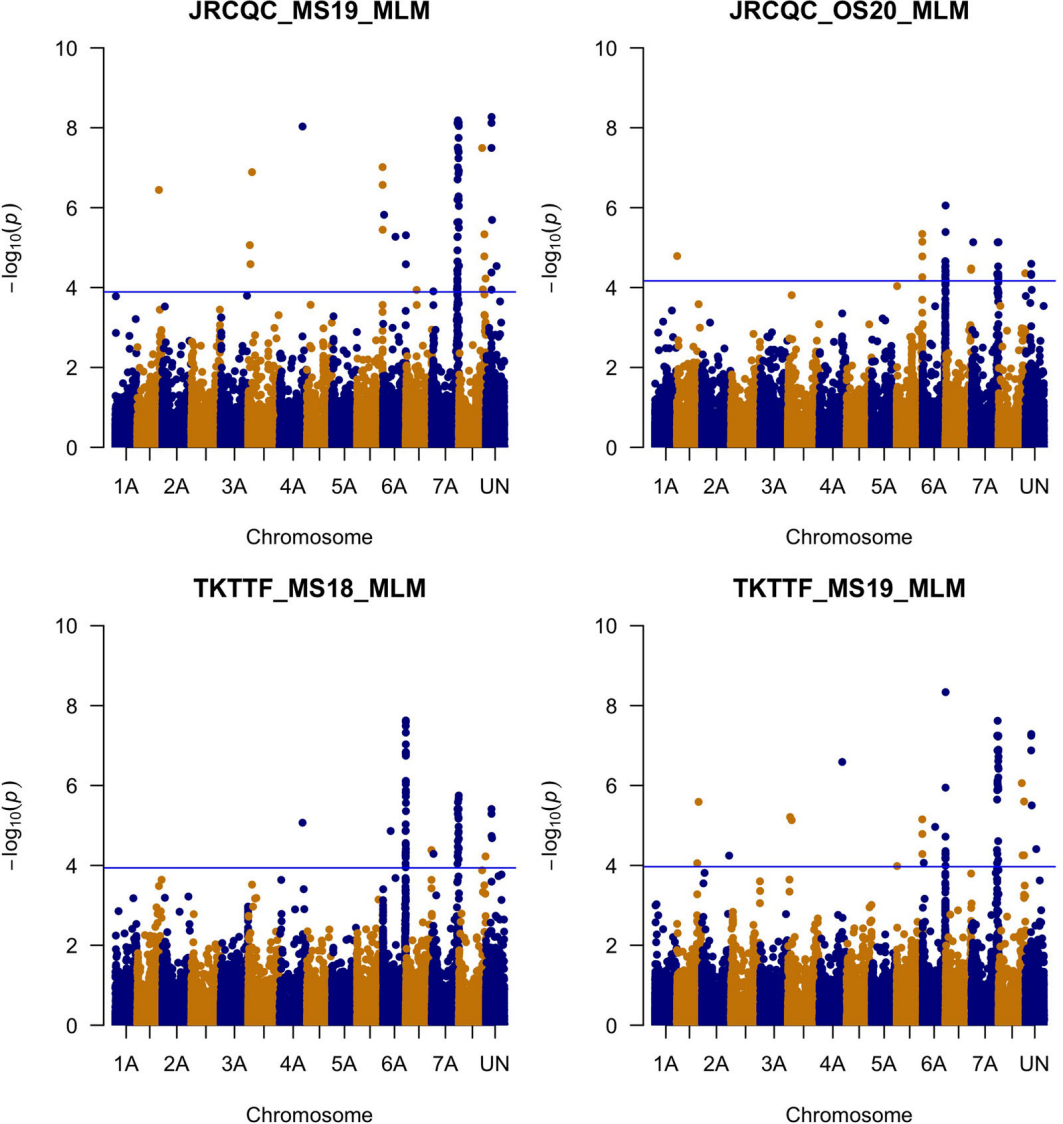
Manhattan plot of genome‐wide association study for field response of durum wheat lines against two *Pgt* races identified using mixed linear model (MLM)

**FIGURE 8:**
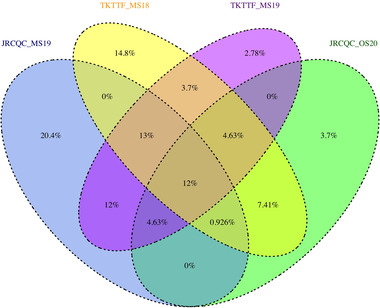
Percentage of common significant markers among field responses of lines against two *Pgt* races across two seasons identified using mixed linear model (MLM)

**FIGURE 9:**
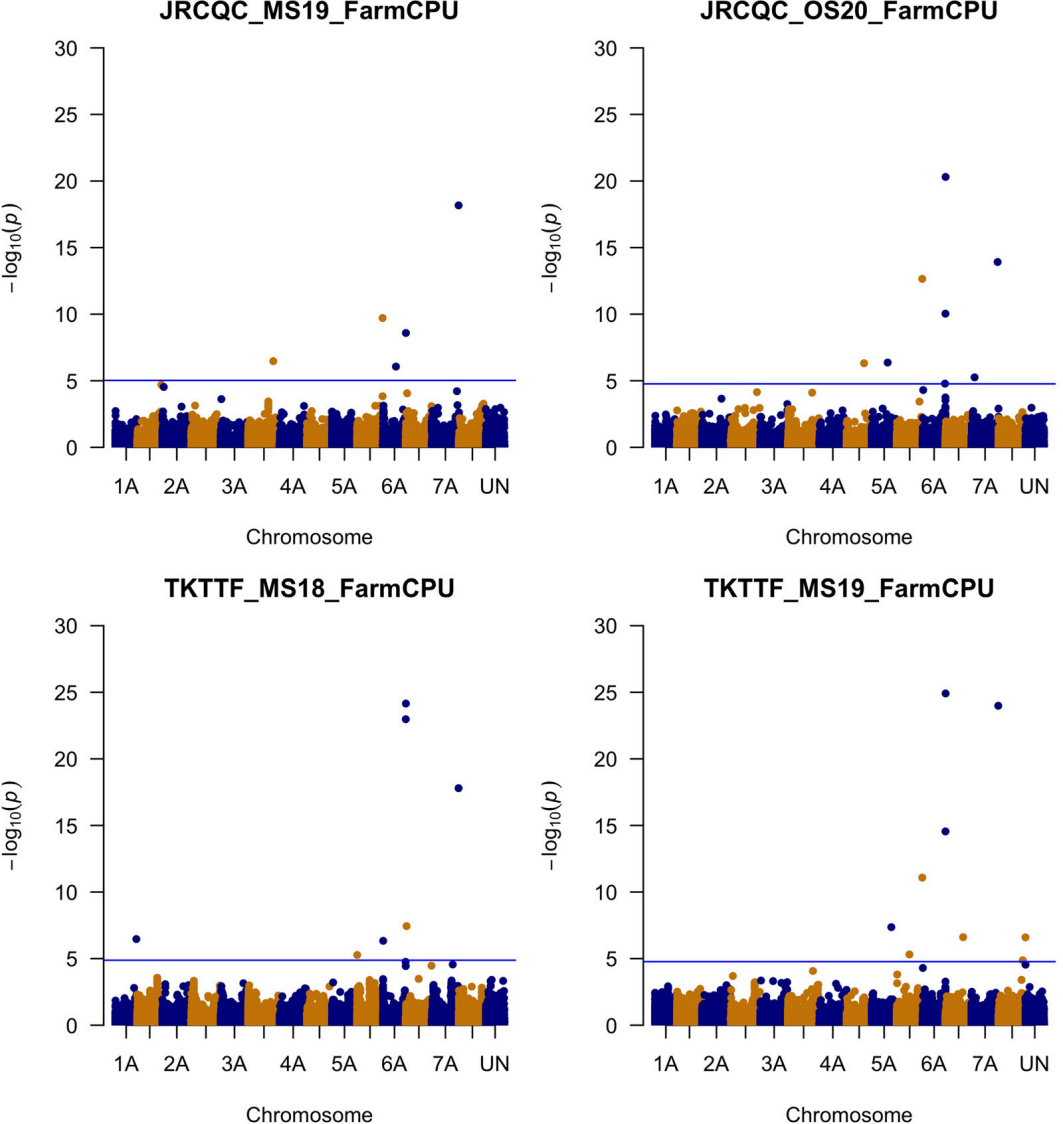
Manhattan plot of genome‐wide association study for field response of durum wheat lines against two *Pgt* races identified using fixed and random model circulating probability unification (FarmCPU)

The BLUPs estimated from the respective models fitted on field responses were used as response variables to fit GWAS models. A total of 108 significant markers distributed on the 14 chromosomes and unaligned contigs were identified using MLM for field resistance against JRCQC and TKTTF across two seasons (Supplemental Table S4, Figure 7). Among the significant markers, 12%, 23.2% and 23.1% were associated with field resistance to four, three and two of the four race‐season combinations, respectively and 41.7% were associated with field resistance to different single race‐season combinations (non‐overlapped region on the Venn diagram) (Figure 8). The consistently significant markers across two to four race‐season combinations were located on chromosomes 1B, 3B, 4A, 5B, 6A, 6B, 7A and on unaligned contigs (Supplemental Table S5). Among the total MTAs identified by MLM, 101 were on known chromosomal regions and grouped into 19 QTL represented by single and multiple nearby markers (Supplemental Table S4; Figure 7). The FarmCPU model identified 19 significant MTAs on nine chromosomes (none on 1B, 2A, 2B, 3Am, and 4A) that were grouped into 16 QTL (Supplemental Table S6; Figure 9). Among those, three QTL on chromosomes 5B (689 Mb), 6A (615 Mb), and 7A (700 and 717 Mb), were consistent between MLM and FarmCPU (Table 4; Supplemental Table S7).

On chromosome 1A, an MTA was identified at 566 Mb for field resistance in TKTTF_MS18 using FarmCPU (Supplemental Table S6; Figure 9). On chromosome 1B, three significant markers (11, 551, and 587 Mb) were identified using MLM. The regions at 11 and 551 Mb were associated with field resistance in JRCQC_OS20 and TKTTF_MS19, respectively. The 587‐Mb locus was associated with field resistance in JRCQC_MS19 and TKTTF_MS19 and it explained 6.7 and 5.7% to the phenotypic variation, respectively (Supplemental Table S4; Figure 7). On chromosome 2A, FarmCPU identified significant MTA at 728 Mb for field resistance in TKTTF_MS19 (Supplemental Table S6).

On chromosome 3B, four significant MTAs (38, 55, 97, and 669 Mb) were identified (Supplemental Tables S4 and S6). The 55 and 97 Mb regions, representing two QTL, were identified using MLM for field resistance in JRCQC_MS19 and TKTTF_MS19. These two QTL explained 11.7 and 10.5% of the phenotypic variation for field response to races JRCQC and TKTTF, respectively (Supplemental Table S4). The MTAs at 38 and 669 Mb regions identified by MLM and FarmCPU, respectively, were associated with field resistance in JRCQC_MS19 (Supplemental Tables S4 and S6).

On chromosome 4A, an MTA at 619 Mb identified by MLM explained 8.6% of the phenotypic variation in JRCQC_MS19 and, on average, 5.9% of the phenotypic variation in TKTTF_MS18 and TKTTF_MS19 (Supplemental Table S4). On chromosome 4B, an MTA at 470 Mb was identified using FarmCPU for field resistance in JRCQC_OS20 (Supplemental Table S4; Figure 9).

On chromosome 5A, MTAs were identified using FarmCPU at 429 and 527 Mb for field resistance in JRCQC_OS20 and TKTTF_MS19, respectively (Supplemental Table S6; Figure 9). Seven significant markers were identified on chromosome 5B using both models. Three MTAs from 689 to 692 Mb (LD, *r* ^2^ = 0.85–0.98) representing the same QTL were consistently identified for field resistance against JRCQC across the two seasons and TKTTF_MS19 (Supplemental Tables S4 and S6). The 689‐Mb locus identified by both MLM and FarmCPU contributed 5.2–7.4% to the phenotypic variation for field response against the two races. Two loci identified by FarmCPU at 7 (TKTTF_MS18) and 345 Mb (TKTTF_MS2019) were associated with field resistance to race TKTTF (Supplemental Tables S4 and S6).

On chromosome 6A, 39 distinct significant markers representing six QTL were identified using MLM and FarmCPU (Supplemental Tables S4 and S6). Five QTL at 5 (TKTTF_MS18), 28 (JRCQC_MS19 and TKTTF_MS19), 205 (TKTTF_MS18), 334 (TKTTF_MS19), and 347 Mb (JRCQC_MS19) were represented by single markers. One QTL represented by 34 significant markers spanning from 603 to 615 Mb explained 3.7–9.1% of the phenotypic variation (Supplemental Tables S4 and S6). For this QTL (603–615 Mb), the marker with the highest contribution to the phenotypic variation was located at 615 Mb (615,604,035 bp) for JRCQC_MS19 (*R* ^2^ = 5.3%), TKTTF_MS19 (*R* ^2^ = 9.1%), and JRCQC_OS20 (*R* ^2^ = 6.5%). This region (615 Mb) was consistently identified by the two models for all race–season combinations and was in LD with markers extending from 612 to 614 Mb and *Sr13* (Table 4; Figure 5). For TKTTF_MS18, a marker at 613 Mb (613,256,520 bp) contributed the most to the phenotypic variation (*R* ^2^ = 8.0%) and the 615 Mb region explained 7.0% of the phenotypic variation (Supplemental Table S4). These two markers (613 and 615 Mb) were in weak LD (*r* ^2^ = 0.13) (Figure 8). On chromosome 6B, FarmCPU identified significant MTAs at 17 and 471 Mb for field resistances in TKTTF_MS18 and TKTTF_MS19, respectively (Supplemental Table S6; Figure 9). In the same chromosome, MLM identified a QTL represented by two significant markers (686 and 687 Mb) for field resistance in TKTTF_MS18 and JRCQC_OS20 and 4.2 and 4.5% of the phenotypic variation, respectively (Supplemental Table S4; Figure 7).

Chromosome 7A harbored the largest number (44) of significant markers representing three putative QTL identified by MLM and FarmCPU (Figures 7 and 9). The MTA at 43 Mb, identified using MLM, was associated with field resistance in JRCQC_OS20 and TKTTF_MS18 (Supplemental Table S4), while the 81‐Mb region, identified using FarmCPU, was associated with field resistance in JRCQC_OS20 (Supplemental Table S6). The remaining 42 MTAs extending from 673 to 727 Mb explained 3.7–8.8% of the phenotypic variation for field responses to races JRCQC and TKTTF across seasons. The markers with the highest contributions to the phenotypic variation were in the 700‐Mb region (700,805,183 bp and 700,727,874 bp; *R* ^2^ = 5.3–8.8%) for field resistance in JRCQC_MS19, JRQC_OS19, and TKTTF_MS19 (Supplemental Tables S4 and S6). For TKTTF_MS18, a significant marker at 721 Mb (721,720,978 bp) contributed the most to the phenotypic variation (*R* ^2^ = 5.8%). This marker (721 Mb) was in strong LD (average *r* ^2^ = 0.88) with the consistently identified significant markers (700 and 717 Mb) by MLM and FarmCPU across all race–season combinations (Figure 6).

On chromosome 7B, seven significant MTAs were identified using MLM and FarmCPU and five of them represent four QTL (Supplemental Tables S4 and S6). A locus at 622 Mb (622,041,448 bp) explained 7.9 and 6.3% of the phenotypic variation in JRCQC_MS19 and TKTTF_MS19, respectively. This marker (622 Mb) was in strong LD (*r* ^2^ = 0.64) with a significant marker at 644 Mb and the two may represent the same QTL. Two MTAs at the 681‐ and 683‐Mb regions were consistently identified in JRCQC_MS19 and TKTTF_MS19 using MLM (Supplemental Table S4). The markers at the 281‐ and 283‐Mb regions were physically close but were not in LD, and the two QTL explained 4.2–5.7% of the phenotypic variation across the two race–season combinations. A QTL at 721 Mb, identified using FarmCPU, was associated with field resistance in TKTTF_MS19 (Supplemental Table S6). Novel loci were consistently identified across races and seasons on chromosomes 3B, 4A, 6A, and 7B. Lines that lack *Sr13* and *Sr58* (*Lr46*), on screening of the same durum panel with KASP markers designed in the genotyping laboratory and previously reported in Megerssa et al. (2020), carried single to multiple favorable alleles at these novel loci (Supplemental Table S8).

## 4 DISCUSSION

The use of genetic resistance is an ecological and economical approach to manage wheat stem rust in different parts of the world. In the current study, we evaluated a panel of spring durum wheat lines representing the CIMMYT durum wheat germplasm pool for the response to four virulent races of the stem rust pathogen (TTKSK, TKTTF, JRCQC, and TTRTF) at the seedling stage and against two of the races (JRCQC and TKTTF) at the adult plant stage. High‐density SNP markers were used to fit three GWAS models (MLM, CMLM, and FarmCPU) and genomic regions associated with seedling and field resistances were identified for future utilization in resistance breeding.

### 4.1 Phenotypic data analysis

#### 4.1.1 Seedling response to the four *Pgt* races

The high frequency of resistant lines and percentage of phenotypic variance explained by the genotypic component (61% for race TTRTF to 91% for race TKTTF) for response to the four races agrees with the qualitative nature of seedling resistance (Supplemental Figure S1; Table 1). However, seedling resistance should be consistent with the field responses to be protective. The relatively lower percentage of lines resistant to races JRCQC (67.1%) and TTRTF (56.4%) compared with races TTKSK (70.6%) and TKTTF (73.1%) is expected because of the documented virulence of the former two races on durum wheat (Olivera et al., 2012b; Olivera Firpo et al., 2019). The seedling resistances observed in the population ranged from single‐ to multiple‐race resistance indicating the effectiveness of the same resistance source against multiple races (Table 2). Our finding of the moderate (0.47) to strong (0.76) correlation among the responses of the lines to the four races further verify this result (Figure 1).

#### 4.1.2 Field response to races JRCQC and TKTTF

Seedling evaluation is the fastest and the cheapest method for screening a large number of lines. However, seedling evaluation should be confirmed by field evaluation for resistance to be reliable. Considering CI ≤ 18 (30MSMR) as resistant in the field, the high frequency of susceptible lines for response to race JRCQC and the low frequency for response to TKTTF (TKTTF_MS18) was not surprising, as JRCQC is more virulent to *Sr13* than TKTTF, which is avirulent on *Sr13* (Supplemental Figure S2). The higher proportion of susceptible lines against race JRCQC compared with race TKTTF (TKTTF_MS18) agrees with the findings of Hundie et al. (2019) on evaluation of 14 durum wheat cultivars against four single races. *Sr13a* is moderately effective against JRCQC; however, the high frequency of susceptible lines to this race could also be explained by the reduced effect of this gene under field conditions (Olivera, et al., 2021), or the temperature dependence of *Sr13* effectiveness as reported by Zhang et al. (2017) in greenhouse evaluation of wheat lines, which may apply in the field because of the expected seasonal variation in temperature. The low frequency of resistant lines in TKTTF_MS19 was unusual, as durum wheat is known to have better resistance against race TKTTF. The lower percentage of phenotypic variance explained by the genotypic component for race JRCQC (59 and 67%) than race TKTTF (74 and 77%) across the two seasons indicates the presence of less variation for resistance to race JRCQC than for TKTTF in the population (Table 3). The moderate correlation between the seedling and field response to race JRCQC (0.37 for MS19 and 0.53 for OS20) and TKTTF (0.55 for MS19 and 0.61 for MS18) may indicate that only some of the lines resistant at the seedling stage are consistently resistant in the field. Thus, the lines that showed consistent resistance in the seedling assay and in the field can be deployed as sources of resistance in durum breeding programs and can also be used for combining with known adult plant resistance genes to increase durability of resistance.

#### 4.1.3 Comparison of identified QTL with previously published studies and known *Sr* genes

Many of the QTL identified in the current study collocated with previously reported QTL markers on tetraploid and hexaploid wheat and cataloged stem rust resistance genes. On chromosome 1A, a QTL at 566 Mb for field resistance in TKTTF_MS18 may tag a region close to regions reported by Edae et al. (2018) (*IWB45411*, 9 Mb away) and Mihalyov et al. (2017) (*IWA4897*, 11 Mb away) (Figure 9; Supplemental Table S4). On chromosome 1B, an MTA at 11 Mb for seedling resistance to race TKTTF and field resistance in JRCQC_OS20 is close to (4.5 Mb away) the *Sr31* locus (Edae & Rouse, 2020). *Sr31* is located on the short arm of chromosome 1B and transferred from rye (*Secale cereale* L.) to hexaploid wheat. This gene had been effective for more than three decades until defeated by the Ug99 race TTKSK (Jin & Singh, 2006; Wanyera et al., 2006). Although *Sr31* is effective against races TKTTF and JRCQC (Olivera et al., 2015), this gene is not expected in the durum panel. So, the 11‐Mb locus is a novel region close to *Sr31*. A region at 22 Mb (22,978,945 bp) associated with seedling resistance against race TTKSK may represent the same region as (2 Mb away) QTL tagging markers *IWB72495* reported by Bajgain et al.(2015b) and *IWA64* reported by Chao et al. (2017) (Figure 4; Supplemental Table S2). A QTL at the 550‐ and 551‐Mb regions for seedling resistance against race TTRTF and field resistance in TKTTF_MS19 collocates with (1–2 Mb away) a QTL linked marker *barc61* reported by Letta et al. (2014) and is expected to be the same QTL. An MTA at 587 Mb for seedling resistance against race TTRTF, field resistance to JRCQC and TKTTF in the main‐season 2019 may map the same region as a QTL tagging marker *IWB40197* (1 Mb away) reported by Edae et al. (2018) (Figures 3 and 7; Supplemental Table S1 and S3). Chromosome 1BL is known to harbor *Sr14* and the pleiotropic APR gene *Sr58* (*Lr46/Yr29/Pm39*) that are known to be effective against several races (McIntosh et al., 1995; Bhavani et al., 2011). However, none of the loci we detected on chromosome 1B are close to markers associated with *Sr14* (*barc8* and *wPt1876*) and *Sr58* (*wmc44)* previously reported by Letta et al. (2013).

A single marker (728 Mb) representing a QTL on chromosome 2A associated with field resistance in TKTTF_MS19 is far away from markers reported by Bajgain et al. (2015b) and Mihalyov et al. (2017) and could be a novel locus (Supplemental Table S3; Figure 7). Chromosome 2A hosts *Sr38* (transferred from *T. ventricosum* (Tausch) Ces. et al.) (Bariana & McIntosh, 1994), which is ineffective against race TKTTF (Olivera et al., 2015; Flath et al., 2018). Eight of the lines in the panel are expected to possess *Sr38* (Ammar, unpublished data, 2020) but this region was undetected because it was below the MAF threshold. On chromosome 2B, a QTL associated with markers ranging from 89 to 97 Mb identified for seedling resistance against race TKTTF may map the same locus as a QTL marker *IWA8599* (1 kb to 7 Mb away) reported by Gao et al. (2017) (Supplemental Tables S1 and S2; Figures 3 and 4).

On chromosome 3A, two QTL identified for seedling resistance to races TTRTF (565 Mb) and TKTTF (565 and 614 Mb) (Supplemental Tables S1 and S2; Figures 3 and 4) were further away from *wmc264* reported by Letta et al. (2014) in the regions of *Sr27* and *Sr35*, and no other nearby regions were previously reported. Moreover, *Sr27* and *Sr35* are orginated from rye and einkorn (*T. monococcum* L.) (McIntosh et al., 1995), respectively, and are unlikely to be present in the durum panel, suggesting that these two QTL are likely novel. On chromosome 3B, no nearby marker is previously reported for loci at 40, 55, 97, and 38 Mb (Supplemental Tables S2–S4; Figures 4, 7, and 9). Chromosome 3BS harbors the known adult plant resistance gene (*Sr2*) that originated from tetraploid emmer wheat [*Triticum turgidum* L. subsp. *dicoccon* (Schrank) Thell.] (McIntosh et al., 1995); however, screening of the panel of lines with an *Sr2* linked marker reported in a different study on the same panel (Megerssa et al., 2020) indicated that this gene was absent in the panel. Therefore, these four QTL are likely to be novel.

An MTA at 17 Mb (17,308,554 bp) on chromosome 4A associated with seedling resistance to race TTRTF co‐locates (789 kb away) with a QTL marker *IWB40004* reported by Bajgain et al. (2015b) (Supplemental Table S1; Figure 3). None of the markers previously reported by several authors (Yu et al., 2011; Letta et al., 2013, 2014; Bajgain et al., 2015b; Chao et al., 2017; Gao et al., 2017) were close to a QTL at the 619‐Mb region of chromosome 4A that was associated with seedling resistance against race TTRTF and field resistance in JRCQC_MS19, TKTTF_MS18, and TKTTF_MS19 (Supplemental Tables S1 and S3; Figures 3 and 7). Therefore, the 619 Mb (619,746,683 bp) locus could be novel for multiple‐race specific resistance including the durum virulent races. A QTL at the 651‐Mb region associated with seedling resistance against race TTKSK maps a region close to a QTL flanking marker (*wPt5857*, 1 Mb away) reported by Yu et al. (2012) and a region associated with *barc78* (4 Mb away) reported by Letta et al. (2014) (Figure 3; Supplemental Table S1). A region at 718 Mb (718944322 bp) associated with seedling resistance to race TKTTF collocates with several markers reported by Bajgain et al. (2015b) including *IWB34733*, *IWB3569*, and *IWB61312* (809 kb away) for seedling resistance of spring wheat collections against TKTTF, marker *IAAV3545* (809 kb) reported by Edae et al. (2018) for seedling resistance of spring wheat against race RCRSC, several markers reported by Edae and Rouse (2020) for resistance of spring wheat against races TKTTF isolate from Ethiopia, (TKKTF‐ETH, the closest marker is 5.6 Mb away) and TTRTF (2 Mb away), marker *IWA4651* (324 kb) linked to *Sr7a* reported by Gao et al. (2017) for seedling resistance of spring wheat against race TTTTF (Figure 4; Supplemental Table S2). Olivera et al. (2015) and Bajgain et al. (2015b) reported that *Sr7a* is effective against race TKTTF isolate from Ethiopia but not against the isolate from Germany (Olivera Firpo et al., 2017). So, based on the proximity to previously reported loci and the race specificity the 718‐Mb region likely maps to the *Sr7a* locus. No markers close to the MTAs at 444 Mb (JRCQC) and 740 Mb (JRCQC_OS20) on chromosome 4B were previously reported. These two loci are possibly novel but they were only identified at the seedling stage and in one season (Supplemental Tables S2 and S4; Figures 4 and 9).

On chromosome 5A, an MTA at 527 Mb associated with field resistance in TKTTF_MS19 may be close to a QTL marker *IWA2836* (9 Mb away) reported by Bajgain et al. (2015b). A QTL linked marker for resistance of spring wheat against race TTRTF reported by Edae and Rouse (2020) match the 581‐Mb locus (5.3 Mb away) associated with seedling resistance to race JRCQC (Supplemental Tables S2 and S4; Figures 4 and 9). A QTL represented by significant markers at 689, 691, and 692 Mb on chromosome 5B collocate with simple sequence repeat markers flanking the region of an all‐stage resistance gene *Sr49* reported by Bansal et al. (2015) (Supplemental Tables S2–S4; Figures 3, 7, and 9). The consistency of this QTL (689–692 Mb) across races (JRCQC and TKTTF), seasons, growth stages (seedling and adult), and the two GWAS models suggests the reliability of the QTL and the association with multiple‐race specific resistance at all growth stages although limited by the low MAF (0.05) (Table 4; Figures 2 and 8). Increasing the frequency of the favorable allele at this locus in the durum breeding lines and incorporating them in future varieties with other resistance genes may prolong the protection against the virulent race JRCQC.

Chromosome 6A harbored six QTL represented by single and multiple markers (Supplemental Tables S1–S4; Figures 3, 4, 7, and 9). A QTL at 5 Mb (5,058,172 bp) region associated with field resistance in TKTTF_MS18 is very close to QTL tagging markers *IWA7913* (138 kb) and *IWB23519* (146 kb) reported by Bajgain et al. (2015b), *IWB72958* (138 kb) reported by Nirmala et al. (2017) as a predictive marker for *Sr8155B1*, markers *IWA7913* (138 kb), and *S6A_PART1_3015737*/*S6A_PART1_3206675* (2 Mb away) associated with *Sr8a* reported by Guerrero‐Chavez et al. (2015) and Edae and Rouse (2020), respectively. *Sr8155B1* is effective against several races but not TTKSK and JRCQC at the seedling stage (Nirmala et al., 2017) and *Sr8a* is ineffective against race TKTTF (Olivera et al., 2015). Thus, the 5‐Mb region likely represents *Sr8155B1* or a new allele of *Sr8* (Supplemental Table S4; Figure 9). No marker close to the QTL at 28, 205, 334, and 347 Mb was previously reported, and these four QTL are likely to be novel. In addition, consistency of the QTL at 28, 205, and 334 Mb across races, races and models, and races, respectively, suggests the reliability of the QTL and the association with multiple‐race specific resistance including the durum virulent race JRCQC (Supplemental Tables S1–S3; Figures 3, 4, and 7). However, further study and validation of these loci is needed. A QTL represented by the markers spanning 602–615 Mb (69 markers) collocated with several previously reported markers in the region of *Sr13* including *CD926040* and *barc104* (Simons et al., 2011; Letta et al., 2013, 2014), *BE471213*, *BE403950*, *CK207347* (Simons et al., 2011; Bhavani et al., 2019), *CJ641478*, *CJ6719993*, and *CJ666008* (Zhang et al., 2017), *IWA4918* (Chao et al., 2017), and *IWA7495* (Simons et al., 2011). Moreover, screening of the same panel of lines with a marker linked to *Sr13* reported in Megerssa et al. (2020) indicated that 69% of the lines in the panel carry *Sr13*. It is known that *Sr13* with its alleles are the mainly used stem rust resistance genes in durum wheat cultivars and germplasm worldwide (Qamar et al., 2009; Olivera et al., 2015; Singh et al., 2015). Different alleles of *Sr13* are expected to be present in the durum panel based on the race specificity and the weak to strong LD with the *Sr13* linked marker (Supplemental Tables S1 and S2; Figure 5). *Sr13a* (R1 and R3 haplotypes in Zhang et al., 2017) is effective against races TTKSK, TKTTF, JRCQC, and TTRTF (Zhang et al., 2017; Olivera Firpo et al., 2019), whereas *Sr13b* (R2 haplotype in Zhang et al., 2017) is effective against the former two races but not against the latter two (Olivera et al, 2012b; Olivera Firpo et al., 2019; Zhang et al., 2017; Randhawa et al., 2018). Accordingly, the SNP at 611 Mb (6A_611495915) that was consistently detected for seedling resistance to the four races may identify allele *Sr13a*. Moreover, a marker at 615 Mb (6A_615604035) was consistent across races TKTTF, JRCQC, and TTRTF at the seedling stage and all race–season combinations in the field. However, differences were observed in the direction of the effect on the response and the allele frequency of markers in LD with 6A_615604035, indicating that this region could be novel or the region of *Sr13a* based on the effectiveness against the four and might be originated from different sources (Supplemental Tables S1, S3, and S4). There was no significant SNP specifically shared between TTKSK and TKTTF only (0%; Figure 2) but based on the race specificity and infection types (IT) on 24 lines we were able to postulate *Sr13b* (Supplemental Table S8). A marker at 612 Mb (6A_612003938) that was identified using FarmCPU for seedling resistance against race TTKSK may map the region of *Sr13b*. The detection of the favorable allele at this locus (6A_612003938) in 18 of the 24 lines that showed low IT to races TTKSK and TKTTF may support our postulation of *Sr13b* (Supplemental Table S8). The identification of three markers (606,107,662; 606,304,231; and 607,001,638 bp) that were in LD with SNPs from 602 to 611 Mb (*Sr13a* region) (Figure 5) for response to JRCQC in the off‐season 2020 only could be in agreement with the results reported by Zhang et al. (2017), which indicated the effectiveness of *Sr13* at high temperature but additional season data is needed to confirm the result. The 615‐Mb (6A_615604035) region identified across all race–season combinations may indicate the effectiveness of the resistance at this locus regardless of the temperature variation in the main and off‐seasons. Nevertheless, the *Sr13* region on chromosome 6A needs further study to survey the presence of other alleles and develop markers that are reliably allele specific.

Several markers (108.9–119 cM) reported by Bajgain et al. (2015b) are very close (195 kb to 4 Mb) to a QTL at the 686‐ and 687‐Mb regions on chromosome 6B (Supplemental Table S3; Figure 7). The closest markers that map the location of *Sr11*, *IWB59175.2* and *IWA4246*, are 195 and 501 kb away from the QTL markers 6B_687598497 and 6B_686489689, respectively. Olivera et al. (2015) reported low infection response (2) of lines carrying *Sr11* against TKTTF and high for JRCQC (3^+^) at the seedling stage, but the MTA we detected was at both growth stages for JRCQC and field resistance against TKTTF (Supplemental Tables S1 and S3). This region is close to the *Sr11* locus but could very well be novel given the known effects of *Sr11*. A QTL at 693 Mb identified for seedling resistance against races TKTTF and TTRTF is close to (492 kb to 1 Mb away) several markers (120.3–122.9 cM) associated with *Sr11* reported by Bajgain et al. (2015b).The closest marker (*IWB46893*) is 492 kb away, suggesting that the 693‐Mb (693,829,939 bp) region may be the *Sr11* locus. Further study on the effectiveness of *Sr11* against the durum virulent race (TTRTF) in the field is needed (Supplemental Table S1; Figure 3).

Chromosome 7A harbored seven QTL represented by single and multiple markers (Supplemental Tables S1–S4; Figures 3, 4, 7, and 9). The QTL markers *wmc479* and *IWA7200* reported by Letta et al. (2013) and Chao et al. (2017), respectively, match loci at 17 Mb (17,624,367 bp, 2 Mb away) associated with seedling resistance to JRCQC and at 67 Mb (6 Mb away) associated with seedling resistance against race TTKSK, respectively (Supplemental Tables S1 and S2; Figures 3 and 4). No QTL marker close to the loci at 43, 51, 81, and 139 Mb has been reported previously but only the 43‐Mb locus could be a true association as it was consistent between JRCQC_OS20 and TKTTF_MS18 (Supplemental Tables S2–S4; Figures 4, 7, and 9). For a QTL represented by the significant markers spanning 668–727 Mb (43 markers), the most significant markers (700 and 717 Mb) that were in LD with the rest of the MTAs collocate with the region of *Sr22* (Figure 6). Markers *IWB5070*, *IWB1874*, *IWB1830*, and *IWB62560* reported by (Bajgain et al., 2015b) are 2 Mb away from the 700‐Mb locus, while *IWB48466* is 5 Mb away from the 717‐Mb region. The origins of *Sr22* are *T. boeoticum* and einkorn (Periyannan et al., 2011) and this gene is effective against several stem rust races including the Ug99 groups of races, JRCQC, TTRTF, and several other races in North America (Olivera et al., 2012b; Olivera Firpo et al., 2019). Similarly, we detected this QTL for seedling resistance against all four races and field resistance against the two races using the two GWAS models (Table 4; Supplemental Table S6). The 721‐Mb region in the same QTL collocates (718 kb away) with a marker in the region of *Sr25* (*BF145935*) (Liu et al., 2010) and 15 lines are known to carry *Sr25* (Ammar, unpublished data, 2020).

On chromosome 7B, a QTL at 622 and 644 Mb identified for seedling resistance against race TTRTF, field resistance in TKTTF_MS19 and JRCQC_MS19 is close to (between 7 and 14 Mb) marker *wmc517* at the *Sr17* locus reported by Letta et al. (2014) (Supplemental Tables S1 and S3). Low IT to race TKTTF (<2^+^) (Olivera et al., 2015) and high IT to race JRCQC (>2^+^) (Olivera et al., 2012b) were reported at the seedling stage on differential lines carrying *Sr17*; however, we detected the association at the adult plant stage for both races, which indicates that the region could be close to *Sr17* but novel. Letta et al. (2013) also reported a QTL flanking marker *wPt4045* as *Sr17* locus and a QTL at 698 Mb identified for seedling resistance against race TKTTF is 873 kb away from this marker and may represent the *Sr17* region (Supplemental Table S2; Figure 3). An MTA at 681 Mb associated with field resistance in TKTTF_MS19 and JRCQC_MS19 is 4 Mb away from a QTL flanking marker *wPt4258* reported by Yu et al. (2014) and may be the same locus. A QTL at 683 Mb (not in LD with 681‐Mb marker) associated with field resistance in TKTTF_MS19 and JRCQC_MS19 may represent the same regions as a QTL identified by markers *wPt1715*, *wPt4298*, and *wPt7191* (3 Mb away) reported by Letta et al. (2013) (Figure 7; Supplemental Table S3). A QTL flanking marker (*wpt8007)* reported by Yu et al. (2014) (2.6 Mb away) and a locus associated with resistance of spring wheat against race TKTTF‐ETH reported by Edae and Rouse (2020) may map the same region as the 721‐Mb locus identified in TKTTF_MS19 (Supplemental Table S4). We were unable to determine the position of nine significant MTAs that were identified on unaligned contigs.

## 5 CONCLUSION

This study revealed that the CIMMYT durum wheat breeding lines harbor race‐specific and multiple‐race resistance to virulent *Pgt* races at the seedling and adult plant stages. Lines consistently resistant in the seedling assay and in the field are being used as sources of resistance in the durum wheat breeding program. We have identified several QTL for resistance to virulent stem rust races at the seedling stage and in the field. Among the 17 QTL identified using MLM for seedling resistance against the four races, eight are putatively novel and among the 20 QTL identified using FarmCPU, 11 are putatively novel. Among the 19 QTL identified using MLM for field resistance against races JRCQC and TKTTF, 12 are putatively novel and among the 16 QTL identified by FarmCPU, seven are putatively novel. Therefore, the stem rust resistance in this study population is controlled by multiple genes. The QTL represented by single markers that were not consistent across races and seasons should be verified before use in future resistance breeding. The markers linked to the six QTL for seedling resistance and three QTL for field resistance that were consistent between the two models can be reliably used in MAS once validated in different populations. Two large‐effect markers in the region of *Sr13* on chromosome 6A that were consistent between races, seasons, and models may identify the *Sr13* haplotypes in different population or *Sr13a* and novel region effective against multiple races. Since the resistance allele at the *Sr49* locus was rare in the population, and this gene is effective against multiple races, this gene should be retained in future selections if no known linkage drag is associated with it. The contribution of the *Sr22/Sr25* region on chromosome 7A to the phenotypic variance was comparable to the *Sr13* region; however, these genes are associated with undesirable agronomic features such as low kernel weight and reduced yield. New recombinant lines less defective in such traits but harboring these genes, either individually or together, are being developed for further evaluation. The evaluation of a panel of lines against virulent races of *Pgt* at the seedling stage and in the field enabled us to identify novel QTL regions specific to the durum virulent races that are consistently identified for other races. Therefore, the novel loci on chromosomes 3B, 4A, 6A, 6B, 7A, and 7B are regions to be validated for use as novel sources of resistance and strategically used in breeding programs. Identification of sources of adult plant resistance is also very important in future resistance breeding of durum wheat against stem rust.

## AUTHOR CONTRIBUTIONS

S. H. Megerssa, M.E. Sorrells and M. Acevedo designed the research; K. Ammar and B. Abeyo developed the mapping population; K. Ammar provided seed to each evaluation site and season; S. H. Megerssa and A.G. Degete carried out the field evaluation; P. Olivera and S. H. Megerssa conducted the seedling evaluation; G. Brown‐Guedira and B. Ward carried out the genotyping and SNP calling; S. H. Megerssa conducted the data analysis and drafted the manuscript; M.E. Sorrells, G. C. Bergstrom, M. Acevedo, P. Olivera and K. Ammar critically reviewed the manuscript.

## CONFLICT OF INTEREST

The authors declare no conflicts of interest.

## Supporting information


**Supplemental Figure S1**. Distribution of seedling responses of durum wheat lines against four *Pgt* races. Data was the linearized scale of the 0–4 IT score to 0–9 scale.
**Supplemental Figure S2**. Distribution of field responses of durum wheat lines against two *Pgt* races. Data was the coefficient of infection (CI). JRCQC_MS19 and JRCQC_OS20 refer to JRCQC main‐season 2019 and off‐season 2020, respectively while TKTTF_MS18 and TKTTF_MS19 refer to TKTTF main‐season 2018 and 2019, respectively.
**Supplemental Table S1**. Lists of durum wheat lines postulated to carry *Sr13b* based on race specificity and lines carrying favorable allele (FA) at the region of *Sr13b* (612003938 bp).
**Supplemental Table S2**. Lists of SNPs significantly associated with seedling resistance to four *Pgt* races identified using MLM. Sheets 1 to 4 correspond to TTKSK, TKTTF, JRCQC and TTRTF, respectively.
**Supplemental Table S3**. Lists of SNPs significantly associated with seedling resistance to four *Pgt* races identified using FarmCPU.
**Supplemental Table S4**. Lists of SNPs significantly associated with field resistance to two *Pgt* races identified using MLM. Sheets 1 to 4 correspond to JRCQC_MS19, JRCQC_OS20, TKTTF_MS18 and TKTTF_MS19.
**Supplemental Table S5**. Lists of common significant markers between races for seedling resistance of lines to the four *Pgt* races and/or between race‐season combinations for field resistance to two *Pgt* races identified using MLM.
**Supplemental Table S6**. Lists of SNPs significantly associated with field resistance to two *Pgt* races identified using FarmCPU.
**Supplemental Table S7**. Lists of common significant markers between races for seedling resistance of a durum wheat panel to the four *Pgt* races and/or between race‐season combinations for field resistance to two *Pgt* races identified using FarmCPU.
**Supplemental Table S8**. Stem rust score of lines negative to *Sr13* and *Sr58* expected to carry the favorable alleles on novel loci on chromosomes 3B, 4A, 6A and 7B that were consistent across races, seasons and/or growth stages.
