# Supplementary material for: Genome‐wide association mapping of seedling and adult plant response to stem rust in a durum wheat panel
Source: Plant Genome. 2021 Jun 19;14(2):e20105. doi: 10.1002/tpg2.20105 (PMC12807237; doi:10.1002/tpg2.20105)
Supplement: Supplementary file 1 [file TPG2-14-e20105-s001.zip › tpg220105-SuppMat-0004.docx]

| **Supplemental Table S3. Lists of SNPs significantly associated with seedling resistance to four *Pgt* races identified using FarmCPU**. | | | | | | | |
| --- | --- | --- | --- | --- | --- | --- | --- |
| **Race** | **Position** | **Chr.** | **P-value** | **Allele** | **AF** | **Effect** | **Proposed gene/allele** |
| TTKSK | 22978945 | 1B | 2.19E-06 | A/**T** | 0.069 | -0.58 | Bajgain et al. (2015); Chao et al. (2017) |
|  | 599292679 | 6A | 5.40E-07 | **G**/C | 0.888 | 0.55 | - |
|  | 611495915 | 6A | 4.80E-28 | **G**/A | 0.850 | 1.53 | *Sr13a* |
|  | 612003938 | 6A | 1.27E-16 | G/**A** | 0.098 | -1.25 | *Sr13b* |
|  | 613294106 | 6A | 8.15E-35 | **C**/T | 0.833 | 1.39 | *Sr13* |
|  | 613748730 | 6A | 8.51E-14 | **T**/A | 0.904 | 1.06 | *Sr13* |
|  | 51332135 | 7A | 1.67E-07 | **A**/T | 0.571 | 0.28 | Likely novel |
|  | 697030516 | 7A | 8.66E-12 | G/**A** | 0.050 | -0.93 | *Sr22* |
| TKTTF | 89523302 | 2B | 2.45E-20 | A/**C** | 0.070 | -1.54 | Gao et al. (2017) |
|  | 614332431 | 3A | 1.31E-06 | C/**T** | 0.058 | -0.68 | Likely novel |
|  | 718944322 | 4A | 2.19E-19 | C/**T** | 0.108 | -1.07 | *Sr7a* |
|  | 691693264 | 5B | 2.34E-08 | T/**A** | 0.050 | -0.99 | *Sr49* |
|  | 609635640 | 6A | 1.91E-14 | **G**/A | 0.851 | 0.85 | *Sr13* |
|  | 611495915 | 6A | 8.07E-06 | **G**/A | 0.851 | 0.88 | *Sr13a* |
|  | 612802438 | 6A | 4.09E-34 | **A**/C | 0.717 | 1.10 | *Sr13* |
|  | 609817335 | 6B | 2.65E-07 | A/**G** | 0.090 | -0.54 | Likely novel |
|  | 698482081 | 7B | 6.54E-07 | **G**/A | 0.867 | 0.45 | Likely *Sr17* |
| JRCQC | 166695897 | 1B | 4.23E-07 | **T**/C | 0.931 | 0.79 | Likely novel |
|  | 40946146 | 3B | 8.80E-06 | **T**/C | 0.576 | 0.30 | Likely novel |
|  | 444117468 | 4B | 6.59E-06 | C/**T** | 0.300 | -0.32 | Likely novel |
|  | 581150219 | 5A | 5.79E-06 | **A**/C | 0.944 | 0.73 | Edae & Rouse (2020) |
|  | 205649407 | 6A | 5.32E-06 | **T**/C | 0.743 | 0.48 | Likely novel |
|  | 611495915 | 6A | 6.53E-27 | **G**/A | 0.850 | 1.60 | *Sr13a* |
|  | 615619215 | 6A | 5.47E-16 | **G**/A | 0.830 | 0.87 | *Sr13* |
|  | 17624367 | 7A | 5.48E-08 | C/**T** | 0.143 | -0.67 | Letta et al. (2013) |
|  | 139258774 | 7A | 1.64E-06 | A/**T** | 0.159 | -0.49 | Likely novel |
|  | 668699732 | 7A | 5.88E-09 | A/**G** | 0.072 | -0.95 | *Sr22* |
|  | 393754818 | UN | 1.16E-05 | G/**A** | 0.054 | -0.83 | - |
| TTRTF | 565464709 | 3A | 1.62E-06 | **G**/A | 0.921 | 0.35 | Likely novel |
|  | 139104893 | 3B | 1.09E-05 | **A**/T | 0.866 | 0.38 | - |
|  | 287211519 | 5B | 2.30E-06 | T/**A** | 0.081 | -0.57 | Likely novel |
|  | 396874801 | 5B | 5.93E-06 | T/**C** | 0.088 | -0.50 | Likely novel |
|  | 609635640 | 6A | 5.75E-10 | **G**/A | 0.848 | 0.59 | *Sr13* |
|  | 612043936 | 6A | 8.29E-06 | T/**C** | 0.303 | -0.25 | *Sr13a/*Novel |
|  | 613131839 | 6A | 8.68E-27 | **G**/A | 0.742 | 0.95 | *Sr13* |
|  | 700805183 | 7A | 2.36E-12 | A/**T** | 0.077 | -0.88 | *Sr22* |
|  | 237571373 | UN | 1.56E-05 | **C**/T | 0.923 | 0.60 | - |
