# Supplementary material for: Genome‐wide association mapping of seedling and adult plant response to stem rust in a durum wheat panel
Source: Plant Genome. 2021 Jun 19;14(2):e20105. doi: 10.1002/tpg2.20105 (PMC12807237; doi:10.1002/tpg2.20105)
Supplement: Supplementary file 1 [file TPG2-14-e20105-s001.zip › tpg220105-SuppMat-0005.docx]

| **Supplemental Table S5. Lists of common significant markers between races for seedling resistance of lines to the four *Pgt* races and/or between race-season combinations for field resistance to two *Pgt* races identified using MLM.** | | | | |
| --- | --- | --- | --- | --- |
| **Position** | **Chr.** | **MAF** | **Seedling resistance to Races** | **Adult plant resistance to race/Season** |
| 11134567 | 1B | 0.054 | TKTTF | JRCQC_OS20 |
| 551557383 | 1B | 0.054 | TTRTF | TKTTF_MS19 |
| 587942809 | 1B | 0.050 | TTRTF | TKTTF_MS19, JRCQC _MS19 |
| 565464709 | 3A | 0.079 | TKTTF, TTRTF | - |
| 55889860 | 3B | 0.064 | - | TKTTF_MS19, JRCQC _MS19 |
| 97870708 | 3B | 0.055 | - | TKTTF_MS19, JRCQC _MS19 |
| 619746683 | 4A | 0.054 | TTRTF | TKTTF_MS18, TKTTF_MS19, JRCQC _MS19 |
| 689821784 | 5B | 0.05 | - | TKTTF_MS19, JRCQC _MS19, JRCQC_OS20 |
| 691693264 | 5B | 0.05 | TKTTF | TKTTF_MS19, JRCQC _MS19, JRCQC_OS20 |
| 692277095 | 5B | 0.05 | - | TKTTF_MS19, JRCQC _MS19, JRCQC_OS20 |
| 28859024 | 6A | 0.05 | - | TKTTF_MS19, JRCQC _MS19 |
| 205649407 | 6A | 0.257 | TTKSK, JRCQC | TKTTF_MS18 |
| 334834338 | 6A | 0.052 | - | TKTTF_MS19, JRCQC _MS19 |
| 603835119 | 6A | 0.204 | JRCQC | TKTTF_MS18 |
| 606107662 | 6A | 0.366 | TTKSK, JRCQC | TKTTF_MS18, JRCQC_OS20 |
| 606107665 | 6A | 0.478 | TTKSK | TKTTF_MS18 |
| 606304231 | 6A | 0.371 | TTKSK | TKTTF_MS18, JRCQC_OS20 |
| 609622362 | 6A | 0.168 | TTKSK, JRCQC | TKTTF_MS18 |
| 609635640 | 6A | 0.150 | TTKSK, JRCQC, TTRTF | TKTTF_MS18 |
| 610129981 | 6A | 0.156 | TTKSK, JRCQC | TKTTF_MS18 |
| 610133407 | 6A | 0.163 | TTKSK, JRCQC, TTRTF | TKTTF_MS18 |
| 610133490 | 6A | 0.159 | TTKSK, JRCQC | TKTTF_MS18 |
| 610146036 | 6A | 0.156 | TTKSK, JRCQC | TKTTF_MS18 |
| 610150266 | 6A | 0.150 | TTKSK, JRCQC | - |
| 610150270 | 6A | 0.154 | TTKSK, JRCQC | - |
| 610150819 | 6A | 0.159 | TTKSK, JRCQC, TTRTF | TKTTF_MS18 |
| 610171399 | 6A | 0.177 | TTKSK, JRCQC, TTRTF | TKTTF_MS18 |
| 610430767 | 6A | 0.145 | TTKSK, JRCQC | - |
| 610475213 | 6A | 0.156 | TTKSK, JRCQC, TTRTF | - |
| 610495870 | 6A | 0.177 | TTKSK, JRCQC, TTRTF | TKTTF_MS18 |
| 611410156 | 6A | 0.085 | TKTTF, TTRTF | - |
| 611495915 | 6A | 0.150 | TTKSK, TKTTF, JRCQC, TTRTF | TKTTF_MS18, TKTTF_MS19 |
| 612003938 | 6A | 0.097 | TKTTF, TTRTF | - |
| 612043936 | 6A | 0.301 | TTKSK, TKTTF, TRTTF | - |
| 612645703 | 6A | 0.083 | TKTTF, TTRTF | - |
| 612802438 | 6A | 0.285 | TTKSK, TKTTF, TRTTF | TKTTF_MS18, TKTTF_MS19, JRCQC _MS19 |
| 612832613 | 6A | 0.260 | TTKSK, TKTTF, TTRTF | TKTTF_MS18, TKTTF_MS19 |
| 612957317 | 6A | 0.264 | TTKSK, TKTTF, TTRTF | TKTTF_MS18, TKTTF_MS19 |
| 613055519 | 6A | 0.262 | TTKSK, TKTTF, TTRTF | TKTTF_MS18, TKTTF_MS19, JRCQC _OS20 |
| 613131839 | 6A | 0.260 | TTKSK, TKTTF, TTRTF | TKTTF_MS18, TKTTF_MS19, JRCQC _OS20 |
| 613194512 | 6A | 0.260 | TTKSK, TKTTF, TTRTF | TKTTF_MS18, TKTTF_MS19 |
| 613217627 | 6A | 0.094 | TKTTF, TTRTF | - |
| 613220409 | 6A | 0.095 | TKTTF, TTRTF | - |
| 613256520 | 6A | 0.273 | TTKSK, TKTTF, TTRTF | TKTTF_MS18, TKTTF_MS19, JRCQC _OS20 |
| 613275023 | 6A | 0.095 | TKTTF, TTRTF | TKTTF_MS18, JRCQC _OS20 |
| 613288180 | 6A | 0.170 | TTKSK, TTRTF | TKTTF_MS18, JRCQC _OS20 |
| 613294096 | 6A | 0.088 | TKTTF, TTRTF | - |
| 613294106 | 6A | 0.167 | TTKSK, TTRTF | TKTTF_MS18, JRCQC _OS20 |
| 613294155 | 6A | 0.264 | TTKSK, TKTTF, TTRTF | TKTTF_MS18, TKTTF_MS19, JRCQC _OS20 |
| 613434999 | 6A | 0.067 | TKTTF, TTRTF | - |
| 613547583 | 6A | 0.168 | TTKSK, TTRTF | TKTTF_MS18, JRCQC _OS20 |
| 613576753 | 6A | 0.099 | TKTTF, TTRTF | - |
| 613576841 | 6A | 0179 | TTKSK, TKTTF, TTRTF | TKTTF_MS18 |
| 613748730 | 6A | 0.095 | TTKSK, TKTTF, TTRTF | - |
| 613908663 | 6A | 0.088 | TKTTF, TTRTF | - |
| 614052038 | 6A | 0.086 | TKTTF, TTRTF | - |
| 614080083 | 6A | 0.247 | TKTTF, JRCQC, TTRTF | TKTTF_MS18, TKTTF_MS19, JRCQC _MS19, JRCQC _OS20 |
| 614329660 | 6A | 0.205 | TTKSK, TTRTF | TKTTF_MS18 |
| 614367995 | 6A | 0.087 | TKTTF, TTRTF | - |
| 614411890 | 6A | 0.105 | TKTTF, TTRTF | - |
| 614784459 | 6A | 0.104 | TKTTF, TTRTF | - |
| 615248120 | 6A | 0.090 | TKTTF, TTRTF | - |
| 615604035 | 6A | 0.279 | TKTTF, JRCQC, TTRTF | TKTTF_MS18, TKTTF_MS19, JRCQC _MS19, JRCQC _OS20 |
| 615604296 | 6A | 0.095 | TKTTF, TTRTF | - |
| 615604386 | 6A | 0.307 | TTKSK, TKTTF, TTRTF | TKTTF_MS18, JRCQC _OS20 |
| 615617605 | 6A | 0.177 | TTKSK, TTRTF | TKTTF_MS18 |
| 615619215 | 6A | 0.173 | TTKSK, TTRTF | TKTTF_MS18 |
| 615636915 | 6A | 0.099 | TTKSK, TTRTF | - |
| 686489689 | 6B | 0.191 | JRCQC | TKTTF_MS18, JRCQC _OS20 |
| 693829939 | 6B | 0.079 | TKTTF, TTRTF | - |
| 43311031 | 7A | 0.092 | - | TKTTF_MS18, JRCQC _OS20 |
| 682951819 | 7A | 0.054 | - | TKTTF_MS19, JRCQC _MS19 |
| 686094342 | 7A | 0.091 | TTRTF | JRCQC _MS19 |
| 686964735 | 7A | 0.075 | - | TKTTF_MS19, JRCQC _MS19 |
| 687410326 | 7A | 0.050 | TTRTF | TKTTF_MS18, TKTTF_MS19, JRCQC _MS19 |
| 690016567 | 7A | 0.052 | TTRTF | TKTTF_MS18, TKTTF_MS19, JRCQC _MS19 |
| 690811708 | 7A | 0.057 | TTRTF | TKTTF_MS18, TKTTF_MS19, JRCQC _MS19, JRCQC _OS20 |
| 690940195 | 7A | 0.057 | TRTTF | TKTTF_MS18, TKTTF_MS19, JRCQC _MS19, JRCQC _OS20 |
| 693915965 | 7A | 0.071 | - | TKTTF_MS19, JRCQC _MS19 |
| 697030510 | 7A | 0.093 | - | TKTTF_MS18, TKTTF_MS19, JRCQC _MS19 |
| 697030516 | 7A | 0.056 | TRTTF | TKTTF_MS18, TKTTF_MS19, JRCQC _MS19, JRCQC _OS20 |
| 698390754 | 7A | 0.096 | - | TKTTF_MS18, TKTTF_MS19, JRCQC _MS19 |
| 700727874 | 7A | 0.059 | TRTTF | TKTTF_MS18, TKTTF_MS19, JRCQC _MS19 |
| 700805183 | 7A | 0.077 | TRTTF | TKTTF_MS18, TKTTF_MS19, JRCQC _MS19, JRCQC _OS20 |
| 710171609 | 7A | 0.055 | - | TKTTF_MS18, TKTTF_MS19, JRCQC _MS19 |
| 714327927 | 7A | 0.067 | - | TKTTF_MS18, TKTTF_MS19, JRCQC _MS19 |
| 714370100 | 7A | 0.052 | - | TKTTF_MS19, JRCQC _MS19, JRCQC _OS20 |
| 714975616 | 7A | 0.091 | - | TKTTF_MS19, JRCQC _MS19, JRCQC _OS20 |
| 717517491 | 7A | 0.056 | TRTTF | TKTTF_MS18, TKTTF_MS19, JRCQC _MS19 |
| 717518884 | 7A | 0.059 | TRTTF | TKTTF_MS18, TKTTF_MS19, JRCQC _MS19, JRCQC _OS20 |
| 718484217 | 7A | 0.098 | - | TKTTF_MS19, JRCQC _MS19, JRCQC _OS20 |
| 719231181 | 7A | 0.064 | - | TKTTF_MS18, TKTTF_MS19, JRCQC _MS19 |
| 719698163 | 7A | 0.057 | TRTTF | TKTTF_MS18, TKTTF_MS19, JRCQC _MS19, JRCQC _OS20 |
| 719787589 | 7A | 0.052 | TRTTF | TKTTF_MS18, TKTTF_MS19, JRCQC _MS19 |
| 721720978 | 7A | 0.065 | TRTTF | TKTTF_MS18, TKTTF_MS19, JRCQC _MS19, JRCQC _OS20 |
| 724668618 | 7A | 0.077 | - | TKTTF_MS18, TKTTF_MS19, JRCQC _MS19, JRCQC _OS20 |
| 724668652 | 7A | 0.052 | - | TKTTF_MS18, TKTTF_MS19, JRCQC _MS19 |
| 681996206 | 7B | 0.063 | - | JRCQC _MS19, TKTTF_MS19 |
| 683438364 | 7B | 0.107 | - | JRCQC _MS19, TKTTF_MS19 |
| 151516737 | UN | 0.123 | TRTTF | JRCQC _MS19 |
| 151742792 | UN | 0.056 | TRTTF | TKTTF_MS18, TKTTF_MS19, JRCQC _MS19, JRCQC _OS20 |
| 151847140 | UN | 0.057 | TRTTF | TKTTF_MS18, TKTTF_MS19, JRCQC _MS19, JRCQC _OS20 |
| 153928527 | UN | 0.056 | TRTTF | TKTTF_MS18, TKTTF_MS19, JRCQC _MS19, JRCQC _OS20 |
| 166522707 | UN | 0.054 | - | TKTTF_MS18, TKTTF_MS19, JRCQC _MS19 |
| 288369273 | UN | 0.075 | - | TKTTF_MS19, JRCQC _MS19 |
