# Supplementary material for: Genome‐wide association mapping of seedling and adult plant response to stem rust in a durum wheat panel
Source: Plant Genome. 2021 Jun 19;14(2):e20105. doi: 10.1002/tpg2.20105 (PMC12807237; doi:10.1002/tpg2.20105)
Supplement: Supplementary file 1 [file TPG2-14-e20105-s001.zip › tpg220105-SuppMat-0006.docx]

| **Supplemental Table S6. Lists of SNPs significantly associated with field resistance to two *Pgt* races identified using FarmCPU.** | | | | | | | |
| --- | --- | --- | --- | --- | --- | --- | --- |
| **Race/Season** | **Position** | **Chr.** | **P-value** | **Allele** | **AF** | **Effect** | **Proposed gene/allele** |
| JRCQC_MS19 | 669183691 | 3B | 3.38E-07 | **C**/G | 0.832 | 2.60 | Bajgain et al. (2015) |
|  | 689821784 | 5B | 1.95E-10 | C/**G** | 0.050 | -6.41 | *Sr49* |
|  | 347960291 | 6A | 8.63E-07 | **C**/T | 0.825 | 2.41 | Likely novel |
|  | 615604035 | 6A | 2.58E-09 | A/**C** | 0.277 | -2.40 | *Sr13* |
|  | 717518884 | 7A | 6.74E-19 | T/**C** | 0.059 | -8.97 | *Sr22* |
| JRCQC_OS20 | 470658058 | 4B | 4.84E-07 | **T**/C | 0.600 | 2.16 | Likely Novel |
|  | 429077415 | 5A | 4.29E-07 | A/**C** | 0.070 | -4.16 | Likely Novel |
|  | 689821784 | 5B | 2.24E-13 | C/**G** | 0.050 | -8.24 | *Sr49* |
|  | 612043936 | 6A | 9.17E-11 | T/**C** | 0.305 | -3.62 | *Sr13* |
|  | 615604035 | 6A | 4.92E-21 | A/**C** | 0.280 | -5.55 | *Sr13* |
|  | 81338498 | 7A | 5.57E-06 | G/**A** | 0.258 | -2.15 | Likely Novel |
|  | 700805183 | 7A | 1.21E-14 | A/**T** | 0.078 | -7.56 | *Sr22* |
| TKTTF_MS18 | 566000158 | 1A | 3.41E-07 | **G**/T | 0.908 | 0.54 | Mihalyove et al. (2017) |
|  | 7669679 | 5B | 5.36E-06 | **G**/A | 0.418 | 0.24 | Likely Novel |
|  | 5058172 | 6A | 4.63E-07 | A/**G** | 0.219 | -0.30 | *Sr8155B1* |
|  | 612043936 | 6A | 1.05E-23 | T/**C** | 0.143 | -0.85 | *Sr13* |
|  | 615604035 | 6A | 7.02E-25 | A/**C** | 0.274 | -0.88 | *Sr13* |
|  | 17572564 | 6B | 3.62E-08 | G/**A** | 0.150 | -0.42 | Yu et al. (2014) |
|  | 717518884 | 7A | 1.59E-18 | T/**C** | 0.060 | -1.06 | *Sr22* |
| TKTTF_MS19 | 527339451 | 5A | 4.36E-08 | A/**G** | 0.111 | -3.87 | Bajgain et al. (2015) |
|  | 345123955 | 5B | 4.90E-06 | A/**C** | 0.193 | -3.04 | Likely Novel |
|  | 689821784 | 5B | 8.29E-12 | C/**G** | 0.050 | -8.45 | *Sr49* |
|  | 612043936 | 6A | 2.81E-15 | T/**C** | 0.146 | -4.86 | *Sr13* |
|  | 615604035 | 6A | 1.22E-25 | A/**C** | 0.277 | -6.64 | *Sr13* |
|  | 471287983 | 6B | 2.47E-07 | C/T | 0.057 | -6.90 | Novel |
|  | 717518884 | 7A | 1.03E-24 | T/C | 0.059 | 12.68 | *Sr22* |
|  | 647958825 | 7B | 1.34E-05 | C/T | 0.082 | -3.89 | - |
|  | 721015179 | 7B | 2.54E-07 | C/T | 0.114 | -3.49 | Yu et al. (2014) |
