# Supplementary material for: Genome‐wide association mapping of seedling and adult plant response to stem rust in a durum wheat panel
Source: Plant Genome. 2021 Jun 19;14(2):e20105. doi: 10.1002/tpg2.20105 (PMC12807237; doi:10.1002/tpg2.20105)
Supplement: Supplementary file 1 [file TPG2-14-e20105-s001.zip › tpg220105-SuppMat-0007.docx]

| **Supplemental Table S7. Lists of common significant markers between races for seedling resistance of a durum wheat panel to the four *Pgt* races and/or between race-season combinations for field resistance to two *Pgt* races identified using FarmCPU.** | | | | |
| --- | --- | --- | --- | --- |
| **Position** | **Chr.** | **MAF** | **Seedling resistance** | **Field resistance** |
| 689821784 | 5B | 0.050 | - | TKTTF_MS19, JRCQC _MS19, JRCQC _OS20 |
| 609635640 | 6A | 0.149 | TKTTF, TTRTF | - |
| 611495915 | 6A | 0.150 | TTKSK, TKTTF, JRCQC | - |
| 612043936 | 6A | 0.301 | TTRTF | TKTTF_MS18, JRCQC _MS19, JRCQC _OS20 |
| 615604035 | 6A | 0.277 | - | TKTTF_MS18, TKTTF_MS19, JRCQC _MS19, JRCQC _OS20 |
| 700805183 | 7A | 0.077 | TTRTF | JRCQC _OS20 |
| 717518884 | 7A | 0.059 | - | TKTTF_MS18, TKTTF_MS19, JRCQC _MS19 |
