# Supplementary material for: Genome‐wide association mapping of seedling and adult plant response to stem rust in a durum wheat panel
Source: Plant Genome. 2021 Jun 19;14(2):e20105. doi: 10.1002/tpg2.20105 (PMC12807237; doi:10.1002/tpg2.20105)
Supplement: Supplementary file 1 [file TPG2-14-e20105-s001.zip › tpg220105-SuppMat-0008.docx]

| **Supplemental Table S1. Lists of durum wheat lines postulated to carry *Sr13b* based on race specificity and lines carrying favorable allele (FA) at the region of *Sr13b* (612003938 bp).** | | | | | | |
| --- | --- | --- | --- | --- | --- | --- |
| **GID** | **REP** | **TTKSK** | **TKTTF** | **JRCQC** | **TTRTF** | **FA** |
| 7145382 | 1 | 2- | 2 | 3+ | 3+ | + |
| 7145526 | 1 | 2- | 2 | 3+ | 3+ | + |
| 7383504 | 1 | 2- | 2- | 3+ | 3+ | + |
| 7383636 | 1 | 2- | 2- | 3+ | 3+ | + |
| 7383851 | 1 | 2- | 1 | 3+ | 4 | - |
| 7383862 | 1 | 3+ | 2 | 3+ | 3+ | + |
| 7407103 | 1 | 2- | 2- | 3+ | 3+ | + |
| 7407855 | 1 | 2- | 2 | 3+ | 2 | + |
| 7409307 | 1 | 2- | 2+ | 3 | 3+ | - |
| 7409435 | 1 | 2- | 2 | 3 | 2- | - |
| 7409461 | 1 | 2- | 2- | 3 | 3+ | + |
| 7409573 | 1 | 2- | 2 | 3+ | 3+ | - |
| 7409575 | 1 | 2- | 2- | 3+ | 3+ | + |
| 7410487 | 1 | 2- | 2 | 3+ | 3+ | + |
| 7410802 | 1 | 2- | 2- | 3+ | 3+ | + |
| 7606753 | 1 | 2- | 2 | 4 | 3+ | + |
| 7606811 | 1 | 2- | 2 | 3+ | 3+ | + |
| 7606825 | 1 | 2- | 2- | 3+ | 3+ | + |
| 7384200 | 1 | 2- | 2 | 3+ | 3+ | + |
| 7384201 | 1 | 2- | 2 | 3+ | 3+ | + |
| 7384216 | 1 | 2 | 2- | 2 | 2 | - |
| 7384219 | 1 | 2- | 2- | 3+ | 3+ | + |
| 7405994 | 1 | 2- | 2+ | 3+ | 3 | + |
| 7406012 | 1 | 2- | NA | 3 | 3 | - |
| 7145382 | 2 | 2- | 2 | 3+ | 3+ | + |
| 7145526 | 2 | 2- | 2 | 3+ | 3 | + |
| 7383504 | 2 | 2- | 2- | 3+ | 3+ | + |
| 7383636 | 2 | 2- | 2- | 3+ | 3+ | + |
| 7383851 | 2 | 2- | 2 | 3+ | 3+ | - |
| 7383862 | 2 | 2- | 2 | 3+ | 3 | + |
| 7407103 | 2 | 2 | 2- | 3+ | 3+ | + |
| 7407855 | 2 | 2- | 2 | 3+ | 4 | + |
| 7409307 | 2 | 2- | 2 | 3+ | 2 | - |
| 7409435 | 2 | 2- | 2 | 2 | 2+ | - |
| 7409461 | 2 | 2 | 2- | 3+ | 3+ | + |
| 7409573 | 2 | 2- | 2- | 3+ | 3+ | - |
| 7409575 | 2 | 2- | 2- | 3+ | 3 | + |
| 7410487 | 2 | 2- | 2- | 3+ | 2 | + |
| 7410802 | 2 | 2- | 2- | 3 | 3+ | + |
| 7606753 | 2 | 2 | 2 | 3+ | 3+ | + |
| 7606811 | 2 | 2- | 2- | 4 | 3+ | + |
| 7606825 | 2 | 2- | 2- | 3+ | 2 | + |
| 7384200 | 2 | 2 | 2- | 3+ | 3+ | + |
| 7384201 | 2 | 2- | 2 | 3+ | 2 | + |
| 7384216 | 2 | 2 | 2 | 3+ | 3+ | - |
| 7384219 | 2 | 2- | 2- | 3+ | 3+ | + |
| 7405994 | 2 | 2- | 2- | 3+ | 2- | + |
| 7406012 | 2 | 2- | NA | 3+ | 2 | - |
