# Supplementary material for: Genome‐wide association mapping of seedling and adult plant response to stem rust in a durum wheat panel
Source: Plant Genome. 2021 Jun 19;14(2):e20105. doi: 10.1002/tpg2.20105 (PMC12807237; doi:10.1002/tpg2.20105)
Supplement: Supplementary file 1 [file TPG2-14-e20105-s001.zip › tpg220105-SuppMat-0009.docx]

| **Supplemental Table S8. Stem rust score of lines negative to *Sr13* and *Sr58* expected to carry the favorable alleles on novel loci on chromosomes 3B, 4A, 6A and 7B that were consistent across races, seasons and/or growth stages.** | | | | | | | | | | | |
| --- | --- | --- | --- | --- | --- | --- | --- | --- | --- | --- | --- |
| **GID** | **REP** | **TTKSK** | **TKTTF** | **JRCQC** | **TTRTF** | **ETOS18** | **ETMS18** | **ETOS19** | **KNMS18** | **KNMS19** | **No. FA at** |
| 6951189 | 1 | 3+ | 4 | 3 | 3+ | 30MS | 50MSS | 40S | 10M | 5MS | 1/6 (3B) |
| **7147178** | **1** | **_** | **1-** | **2-** | **2-** | **10RMR** | **0** | **0** | **0** | **5R** | **5 / 6** |
| **7147177** | **1** | **_** | **;** | **2-** | **2-** | **10MRR** | **0** | **0** | **0** | **5R** | **6/6** |
| 7384262 | 1 | 3+ | _ | 3+ | 3 | 70S | 50S | 50S | 40MSS | 50MSS | 1 /6 (3B) |
| **6469777** | **1** | **2-** | **2-** | **2-** | **2** | **10MR** | **5MR** | **10MRMS** | **10M** | **10MS** | **1 /6 4A** |
| **6951159** | **1** | **2-** | **2-** | **2-** | **2-** | **15SMS** | **_** | **40MSS** | **5M** | **10MS** | **2 /6 (6A &7B)** |
| 7606790 | 1 | 3+ | 3+ | 3 | 3 | 15SMS | 70S | 40S | 50M | 50MSS | 1 /6 (7B) |
| 6951189 | 2 | 3+ | 3+ | ; | 3+ | 30SMS | 20MSMR | 30MS | 10M | 30M |  |
| **7147178** | **2** | **_** | **;** | **2-** | **2-** | **5RMR** | **0** | **TMR** | **TR** | **5R** |  |
| **7147177** | **2** | **_** | **;** | **2-** | **;** | **TR** | **0** | **0** | **5R** | **10RMR** |  |
| 7384262 | 2 | 3+ | _ | 3+ | 3 | 50S | 50S | 50S | 70MSS | 50MSS |  |
| **6469777** | **2** | **2-** | **2-** | **2-** | **2** | **10MR** | **20MSMR** | **50S** | **10RMR** | **5R** |  |
| **6951159** | **2** | **2-** | **2** | **2-** | **2-** | **20MSS** | **10MSMR** | **30MSS** | **5RMR** | **20M** |  |
| 7606790 | 2 | 3+ | 3+ | 3+ | 3 | 60S | 40S | 40S | 50M | 50MSS |  |
| Bold face indicate lines showed resistance at all stage | | | | | | | | | | | |
